# Systematics and phylogeography of bats of the genus *Rhynchonycteris* (Chiroptera: Emballonuridae): Integrating molecular phylogenetics, ecological niche modeling and morphometric data

**DOI:** 10.1371/journal.pone.0285271

**Published:** 2023-05-04

**Authors:** Alejandro José Biganzoli-Rangel, Omar Daniel Leon-Alvarado, Lizandra Jaqueline Robe, María Angélica Meza, Eliécer Eduardo Gutiérrez, Andressa Paladini

**Affiliations:** 1 Laboratorio de Sistematica, Entomologia e Biogeografia, Programa de Pós-Graduação em Biodiversidade Animal, Universidade Federal de Santa Maria, Santa Maria, Rio Grande do Sul, Brazil; 2 Programa de Pós-Graduação em Biodiversidade Animal, Universidade Federal de Santa Maria, Santa Maria, Rio Grande do Sul, Brazil; 3 Universidad Industrial de Santander, Ciudad Universitaria, Bucaramanga, Santander, Colombia; 4 Departamento de Zoologia, Universidade Federal do Paraná, Curitiba, Paraná, Brazil; National and Kapodistrian University of Athens, GREECE

## Abstract

*Rhynchonycteris* is a monotypic genus of Embalonurid bats, whose geographic distribution extends from southern Mexico to tropical regions of the South American continent, including Trinidad and Tobago. Although species that have a wide geographic distribution are frequently revealed to be polytypic, to date, no study has evaluated the taxonomic status of populations of *Rhynchonycteris naso*. Thus, the aim of this study is to address the patterns of phylogeographic structure and taxonomic subdivision of *R*. *naso* using molecular phylogenetics, morphometric data and ecological niche modeling. Phylogenetic results recovered using the genes *COI*, *Cytb*, *Chd1*, *Dby*, and Us*p9x*, supported the monophyly of the genus *Rhynchonycteris*, in addition, a deep phylogeographic structure was revealed by the mitochondrial gene *COI* for the populations of Belize and Panama in comparison to those of South America. The PCA, and the linear morphometry indicated an apparent differentiation between the cis-Andean and trans-Andean populations. Furthermore, according to the skull morphology, at least two morphotypes were identified. Ecological niche modeling projections in the present have shown that the Andean cordillera acts as a climatic barrier between these two populations, with the depression of Yaracuy (Northwest Venezuela) being the only putative climatically suitable path that could communicate these two populations. On the other hand, projections for the last glacial maximum showed a drastic decrease in climatically suitable areas for the species, suggesting that cycles of lower temperatures played an important role in the separation of these populations.

## Introduction

Bats of the Emballonuridae family are currently recognized by eight genera and 22 species in the New World, and five genera and 30 species in the Old World [1–3]. These strictly insectivorous bats are found in tropical and subtropical areas, where they provide important ecosystem services, acting in the control of insect populations [4–6]. Its wide geographic distribution in the Neotropics extends from southern Mexico, central America and central South America, and into the Old World across Africa, Madagascar, South Asia, part of Australia and the Pacific Islands from eastern Samoa [3].

Within the New World Emballonurids, *Rhynchonycteris* W. Peters, 1867 [7] is a monotypic genus of bats presenting a neotropical distribution [8]. These bats generally inhabit mangroves and lowland forests, close to streams, rivers and lakes [9], where they occur from 0 to 900 meters above sea level (m.a.s.l.) [8,10]. Their distribution extends from southern Mexico, through Central America, eastern Peru, northern Bolivia and central Brazil, including Trinidad and Tobago [1,8,10–13]. A striking feature of the wide geographic distribution of *Rhynchonycteris naso* (Wied-Neuwied, 1820) [14] is the division of these populations by the Andes into two large groups: the trans-Andean, occurring north of the Andean slopes, and the cis-Andean, distributed in the southern part of the Andean slopes [15].

Several studies have investigated the phylogenetic relationships within the Emballonuridae family. These studies evaluated skull morphology [16], protein electrophoresis [17], hyoid bone morphology [18], morphology and behavior [19] and molecular analysis with nuclear and mitochondrial genes [20]. Nevertheless, none of these studies tested the monophyly of *Rhynchonycteris naso* with a comprehensive sampling of terminal taxa or evaluated differences among its populations taking into account its biogeography, ecology and phylogeography.

Currently, due to the ubiquity of cryptic diversification, the discovery of new species is rarely related to morphological variation. In fact, new species are usually delimitated by integrating multiple approaches, as behavior, ecology and molecular markers [21], with subtle or unnoticed morphological differences. This is why, for example, in other genera of bats, genetic tools have helped to uncover cryptic diversity and to clarify interspecific relationships [22–24]. Other approaches as Ecological Niche Models (ENMs) also frequently contribute to reduce the Linnean and Wallacean impediments [25,26], related to shortages in the knowledge about the number and the distribution of most species, respectively.

Due to the lack of studies assessing the monophyly and the taxonomic status of different populations of *Rhynchonycteris naso*, the aim of this study is to address the patterns of distribution of genetic and morphological variation of this species. Furthermore, we evaluated geographic and non-geographic morphometric variation. Finally, we assessed whether ENM projections performed for the species in various past climate scenarios are congruent with population divergence patterns.

## Materials and methods

### Taxonomic sampling

To test the monophyly of *R*. *naso*, we selected DNA sequences available in GenBank from all recognized neotropical species of the Emballonuridae family [1,2]. The ingroup was composed by 20 species (*Balantiopteryx infusca*, *Balantiopteryx io*, *Balantiopteryx plicata*, *Centronycteris centralis*, *Centronycteris maximiliani*, *Cormura brevirostris*, *Cyttarops alecto*, *Diclidurus albus*, *Diclidurus ingens*, *Diclidurus isabellus*, *Diclidurus scutatus*, *Peropteryx kappleri*, *Peropteryx leucoptera*, *Peropteryx macrotis*, *Peropteryx pallidoptera*, *Peropteryx trinitatis*, *Saccopteryx bilineata*, *Saccopteryx canescens*, *Saccopteryx gymnura* and *Saccopteryx leptura*) in addition to *R*. *naso*. The only species of neotropical Emballonuridae that could not be included in the analysis due to the absence of any sequence is *Saccopteryx antioquensis*, endemic to the northern Andes of Colombia [27].

Furthermore, we included sequences of six Old World embalonurids as an outgroup (*Emballonura beccarii*, *Emballonura monticola*, *Emballonura raffrayana*, *Mosia nigrecens*, *Taphozous melanopogon* and *Taphozous longimanus*). These species encompass the group most closely related to New World embalonurids [20,28].

### Genetic data

For the phylogenetic analysis we used mitochondrial and nuclear (autosomal and sexual) gene sequences available in GenBank. These sequences included fragments of Cytochrome C Oxidase I (*COI*), Cytochrome b (*Cytb*), Chromodomain-Helicase-DNA-Binding Protein 1 (*Chd1*), DEAD-Box Helicase 3 Y-Linked (*Dby*), and Ubiquitin Specific Peptidase 9 X-Linked (*Usp9x*) (see [20]) (for accession numbers, see S1 Table).

### Phylogenetic analysis

We aligned the sequences of each gene using the MUSCLE algorithm (K-mer distance) [29] implemented in MEGA X [30], with default options. Individual gene trees were reconstructed by maximum likelihood (ML) in iqTree v.2 [31], using ModelFinder [32] to select the best substitution model. Clade support was measured through 1000 ultrafast bootstrap replicates [33]. A partitioned Bayesian inference analysis was performed in MrBayes v3.2.2 [34] and implemented in the Cipres Science Gateway v3.3. The best nucleotide evolution model was selected for each gene using jModelTest2 [35,36], using the Akaike Information Criterion (AIC) [37]. The Bayesian analysis consisted of a cold chain and three heated chains, using a sampling approach by the Markov Chain Monte Carlo (MCMC) algorithm, with 50,000,000 generations, sampling every 5,000 generations. The initial 25% of the samples were discarded as burn-in. Samples from each run were evaluated in Tracer [38] and a cutoff point of 200 for the effective sample size (ESS) was used to determine if the Markov chain reached stationarity and converged. Finally, the majority-rule tree was rooted in *Taphozous longimanus*.

### Phylogeographic structure

To assess the phylogeographic structure of *R*. *naso*, we used *COI* sequences available for the species in GenBank. The haplotypes and their frequencies were determined using the software DnaSP v6.0 [39]. Pairwise Φ_ST_ values among populations were estimated in Arlequin v3.5 [40], with significance inferred with 10,000 permutations. A Mantel test was then performed to assess whether spatial distance (Euclidean geographic distance) is correlated with genetic distance, in an isolation by distance pattern.

Data were also analyzed using the software SAMOVA v1.0 [41], which defines the best group of populations (*K*) that are geographically homogeneous but genetically differentiated. To this task, *K* values ranging from 2 to 10 were evaluated with 100 initial conditions. The *K* value that produced the highest F_ct_ was determined, and individuals were assigned to groups following this classification. The general structure of the population was then inferred with an Analysis of Molecular Variance (AMOVA). Finally, median-joining haplotype networks [42] for the main haplogroups were generated using the software Network, v10 (http://www.fluxus-engineering.com/).

### Genetic distance

Pairwise intraspecific and interspecific genetic distances of the *COI* gene were calculated in the MEGA X software [30] using two approaches: Kimura 2 parameters substitution model and *p*-distance.

### Morphometric analysis

Museum acronyms for specimens examined are: AMNH, American Museum of Natural History, New York, USA; IAVH, Instituto Alexander Von Humboldt, Bogotá, Colombia; ICN, Instituto de Ciencias Naturales Universidad Nacional de Colombia, Bogotá, Colombia; UIS, Universidad Industrial de Santander, Bucaramanga, Colombia; MUSM, Museo Universidad Nacional Mayor de San Marcos, Lima, Peru; MZUSP, Museu de Zoologia da Universidade de São Paulo, São Paulo, Brazil; UFES, Museu da Universidade Federal de Espírito Santo, Espírito Santo, Brazil; QCAZ, Museo de Zoología de la Pontificia Universidad Católica del Ecuador, Quito, Ecuador.

A digital caliper with an accuracy of 0.01 mm was used to take one external (forearm) and 10 craniodental measurements of 121 specimens, identified according to Hood and Garner [12]. The measurements were previously used in other studies of the Emballonuridae family [8,43]. The craniodental, and external measurements used in this study were:

**Greatest length of skull (GLS)**: Distance from the posteriormost point on the occiput to the anteriormost point on the premaxilla (excluding the incisors).
**Condilobasal length (CBL)**: Distance between a line connecting the posteriormost margins of the occipital condyles and the anteriormost point on the premaxilla.
**Height upper canine (UP CANIN)**: Greatest length from point immediately dorsal to cingulum to end of tooth.
**Breadth braincase (BR BCASE)**: Breadth just dorsal to posterior juncture of zygomatic process.
**Breadth at mastoids (MASTOID)**: Greatest breadth at mastoid processes.
**Maxillary toothrow (MTR)**: Length from anterior alveolar border of canine to posterior alveolar border of M^3^.
**Width at upper molars (M3 M3)**: Width between alveolar borders of upper third molars.
**Width at upper canines (C1 C1)**: Width between alveolar borders of upper canines.
**Dentary length (DENT LEN)**: From midpoint of mandibular condyle to anteriormost point of dentary.
**Maxillary toothrow (MTR)**: Length from anterior alveolar border of canine to posterior alveolar border of M^3^.
**Forearm length (FA)**: Distance from the elbow (tip of the olecranon process) to the wrist (including the carpals).

Some structures could not be measured due to skull deterioration. Therefore, some data were inferred with the *missMDA* package [44] applied in R v3.6.1 [45].

All measurements were transformed into log base 10. Morphometric differences between populations of *R*. *naso* and between sexes were evaluated using a Principal Component Analysis (PCA) based on the variance-covariance matrix. To show the relationships between groups in the morphospace, the values of the principal components (PC) were graphically represented. Furthermore, statistical differences between sexes and between the cis-Andean and trans-Andean populations were evaluated with a Multivariate Permutation Analysis of Variance (PERMANOVA). The methods were applied in R v3.6.1 [45].

Finally, the skulls were photographed with a Nikon D3500 with an AF-S 40mm 1:2.8G Nikkor Micro lens. The skulls were photographed in ventral, dorsal and lateral views to assess possible differences in morphology.

### Ecological niche modeling

To assess whether the climate suitability model projections to past climate scenarios are congruent with the inferred divergence patterns, we build the ecological niche models (ENM) using the maximum entropy algorithm implemented in Maxent. v4.0 [46]. A total of 3904 occurrences of *R*. *naso* were obtained from Global Biodiversity Information Facility—GBIF (http://www.gbif.org). Analyzes were based exclusively on data from voucher specimens deposited in natural history museums. Occurrences without location-specific information were removed, as well as occurrences without geographic uncertainty or with uncertainty > 5000 meters. To reduce the sampling bias that may exist and the spatial autocorrelation of the data [47], we used the *spThin* package [48] in R v3.6.1, so that samples have a minimum distance of 10 kilometers between each other (see [49,50]). Thus, we got 227 filtered occurrences, divided into 170 calibration or training points and 57 test points.

To create the models and effectively infer areas of climate suitability for the target organism, the environmental data was composed of 19 bioclimatic layers (spatial resolution = 2.5’, which corresponds to the uncertainty inherent in the occurrence data) downloaded from WorldClim v1.4 [51]. Four bioclimatic variables (BIO7, BIO8, BIO18, BIO19) were excluded from the analysis because these variables include known spatial artifacts [52]. Four sets of environmental variables were chosen, based on jackknife tests applied in Maxent. Variables that contributed the least were sequentially removed. The sets used for the analyzes consisted of 15, 8, 6, and 4 variables.

The models were calibrated in the known range of distribution for the species (from southern Mexico to central South America), which is considered a working hypothesis of M for the species [53]. To acquire an adequate representation of the environments available for these species, 20,000 random background points were included in the delimited study area.

Models were created with 10 bootstrap replicates with a variety of different combinations of feature classes (Linear (L), Quadratic (Q), Product (P), Threshold (T), and Hinge (H)), and 18 regularization multiplier values (0.1–1 with intervals of 0.1; 1–6 with intervals of 1; 8, and 10). Model performance was evaluated considering statistical significance (partial ROC (Receiver Operating Curve); [54]), predictive power (omission rates, *E* = 5%, [55]), and complexity level (AICc; [56]), in this order.

After calibrating and selecting the models, model projections were performed to present and past scenarios. Model projections involves transferring the niche models to a different space than the one used for model calibration; a Mobility-Oriented Parity analysis (MOP) was performed to quantify the similarity between the calibration and transfer regions [57].

Past environmental data were characterized for the Last Interglacial (approx. 120,000–140,000 years ago), whose conditions were obtained from the PaleoClim [58,59], and for the Last Glacial Maximum (approx. 22,000 years ago), and the Mid Holocene (approx. 6,000 years ago), whose conditions were obtained from WorldClim [51]. The Last Glacial Maximum and the Mid Holocene periods were evaluated under three different climate models (GCMs; MPI-ESM-P, CCSM4 and MIROC-ESM).

To identify and graphically represent changes in suitability, and changes in suitable areas in past scenarios, the approach of Cobos *et al*., [60] was used. All analyzes were performed in R v3.6.1 with the *kuenm* package [61].

In addition to the models generated considering the total distribution of the species (hereafter called the “Total model”), we generated models for the “cis-Andean” (85 occurrences to calibrate and 32 occurrences to test the models), and “trans-Andean” populations (85 occurrences to calibrate and 25 occurrences to test the models). The methodology remained the same as described above.

## Results

### Phylogenetic analysis

**Fig 1:**
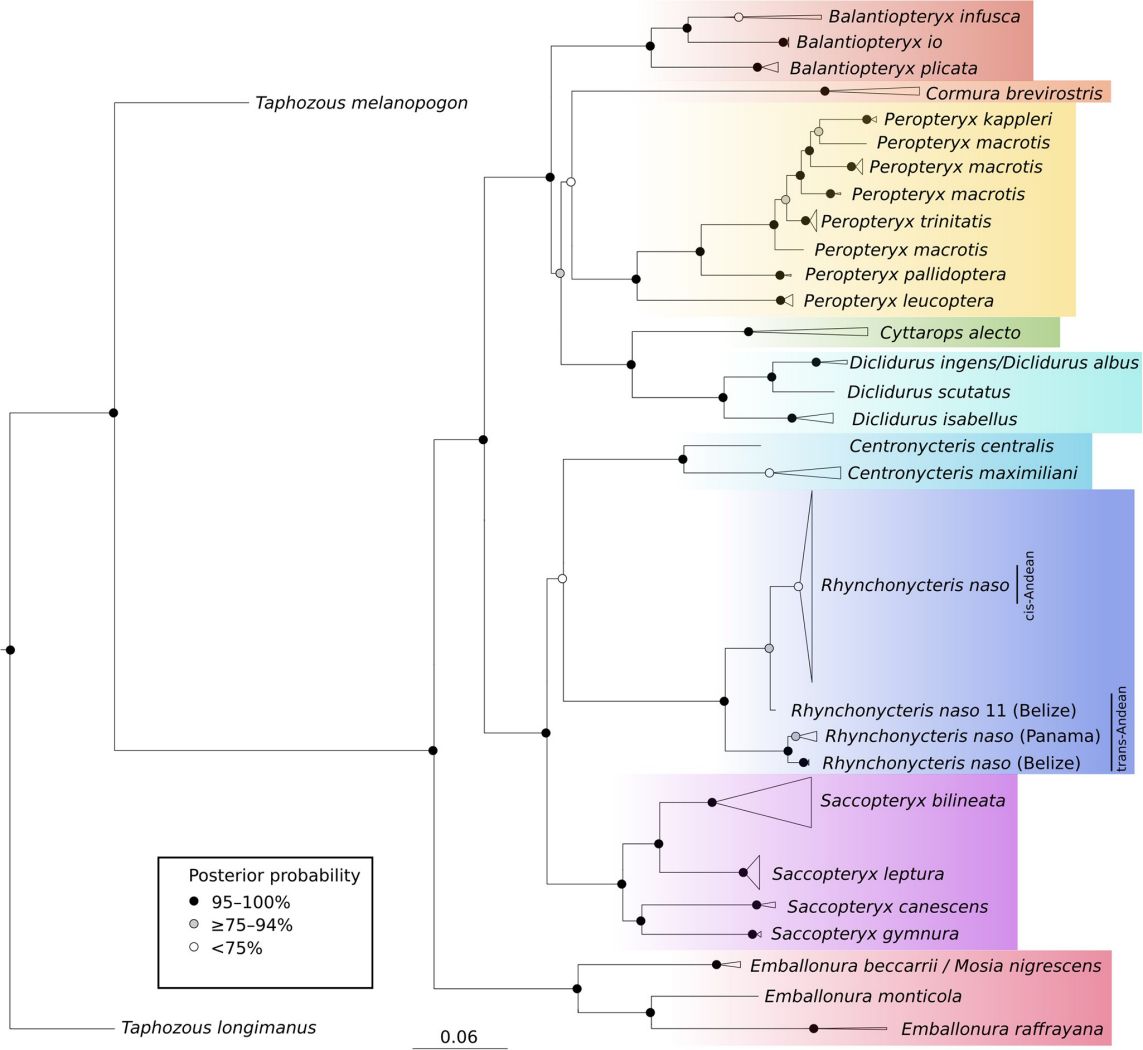
Majority-rule tree inferred from the combined data set of embalonurid bats through a partitioned Bayesian analysis. Bayesian posterior probabilities (> 50%) are indicated at each node with color dots. Branch lengths are proportional to the scale, given in substitutions per nucleotide.

The multi-locus dataset encompassed 3521 nucleotides, characterized for a total set of 249/27 ingroup specimens/species. The selected nucleotide substitution models were: HKY + I for *Chd1* (423 nucleotides in length), GTR + I + Γ for *COI* (658 nucleotides), HKY + I + Γ for *Cytb* (1140 nucleotides), GTR + I for *Dby* (729 nucleotides), and HKY + I for *Usp9x* (571 nucleotides). The Bayesian analysis recovered *Rhynchonycteris naso* as a monophyletic group with a high Bayesian posterior probability (100%), a result that was also supported by individual gene trees (S3 Fig). The target species was positioned as a sister group of the *Centronycteris* genera, but the clade formed by these two groups had a low posterior probability (72%) (Fig 1), and was only supported by the *Dby* gene tree. Our analysis also suggested a geographic structure for the populations of *R*. *naso*, with populations from South America (cis-Andean) being recovered as monophyletic in regard to those from Belize and Panama (trans-Andean), with high posterior probability (99%) (Fig 1), and high bootstrap value (100%) in the *COI* gene tree (S3 Fig). Nevertheless, in the concatenated analysis, the trans-Andean specimens were recovered as a grade, possibly because of shortages related to missing data. In fact, most individuals from Belize and individuals from Panama presenting only *COI* sequences were recovered as a clade, whereas “*Rhynchonycteris naso* 11*”* from Belize was represented only by *Chd1* sequences and appeared as a sister group of the cis-Andean specimens with low posterior probability (76%).

### Phylogographic structure

**Fig 2:**
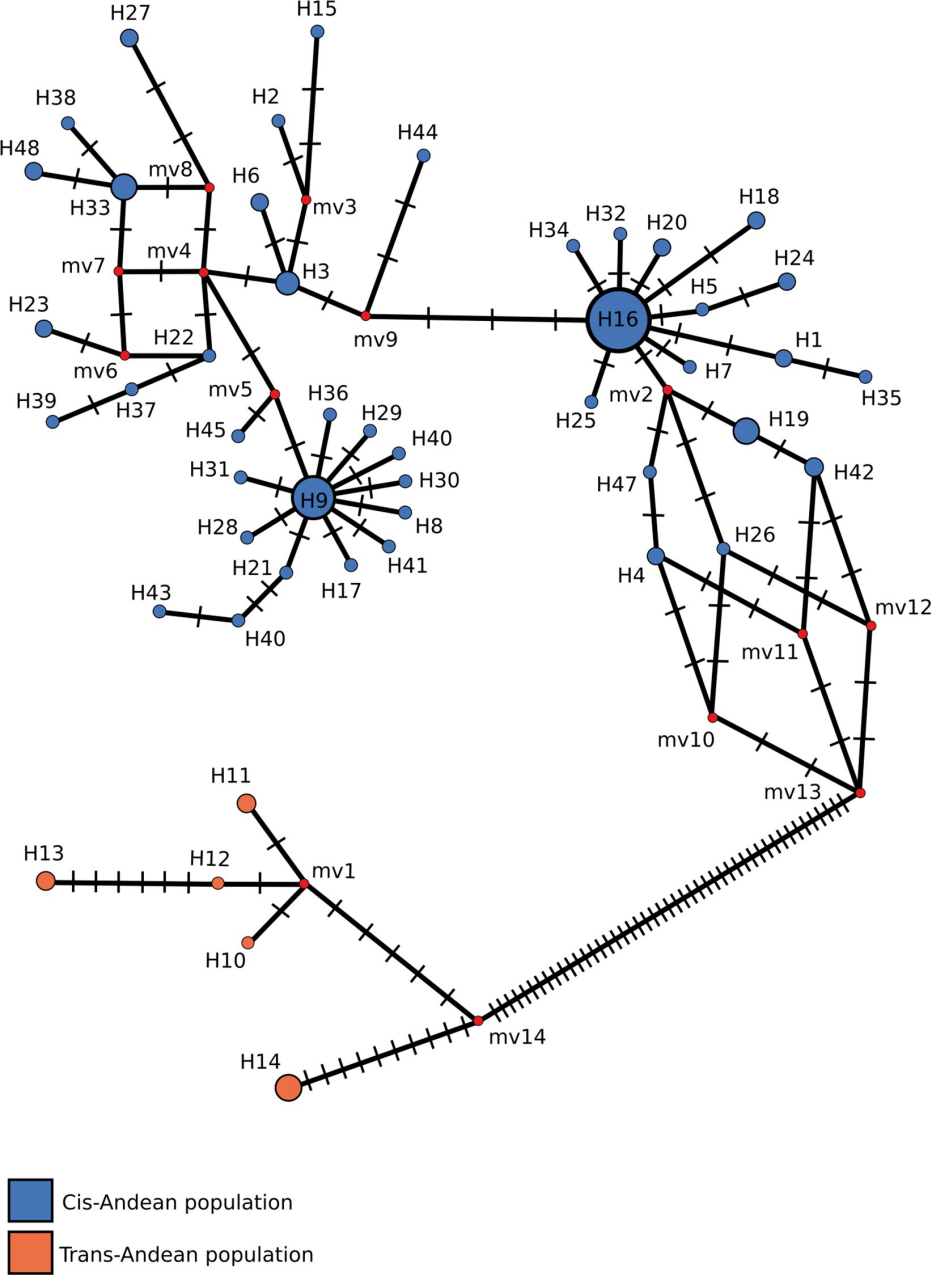
Median-joining haplotype network of the *COI* sequences recovered for *Rhynchonycteris naso*. Each circle represents a different haplotype, whose size is proportional to frequency. Colors subdivide haplotypes according to their sampling points as shown by the legend. Red small circles represent median vectors, and dashes in the lines connecting different haplotypes represent the number of substitutions between them.

Out of a total of 106 *COI* sequences recovered for *R*. *naso*, 104 were evaluated for the presence of phylogeographic structure, and two sequences were excluded due to the high number of missing nucleotides. These resulted in 48 different haplotypes (S2 Table), none of which was shared between trans-Andean and cis-Andean populations. In fact, these groups encompassed different haplogroups in the network, differing by at least 47 substitutions (Fig 2). Furthermore, the populations of Belize (Hap14) and Panama (Hap10, Hap11, Hap12 and Hap13) differed by at least 15 substitutions.

All the 10 groups evaluated with SAMOVA showed significant differences between them (*P* < 0.001) (Table 1). The highest value of Φ_CT_ was reached with *K* = 3 groups (Φ_CT_ = 0.90809) and presented a subdivision among the population of Belize (harboring haplotype Hap14), the population of Panama (harboring haplotypes Hap10, Hap11, Hap12 and Hap13), and a third group including all other populations (see S2 Table for nomenclature). Thus, SAMOVA analysis support the hypothesis obtained with the phylogeny results (Fig 1), suggesting trans-Andean and cis-Andean populations of *R*. *naso* are deeply differentiated.

**Table 1:** SAMOVA analysis performed with 34 populations of *Rhynchonycteris naso* (see S2 Table), using the gen *COI*.

*K*	Group composition	Φ_CT_	Φ_SC_	Φ_ST_
2	(S4,S12,S24,S28); other populations	0.90253***	0.04701***	0.90711***
3	(S4,S12,S28); (S24); other populations	0.90809***	0.00448***	0.9085***
4	(S4); (S12,S28); (S24); other populations	0.90751***	0.0035***	0.90783***
5	(S3); (S4); (S12,S28); (S24); other populations	0.89528***	-0.00341***	0.89492***
6	(S3); (S22); (S24); (S30); (S4,S12,S28); other populations	0.87117***	-0.00684***	0.87089***
7	(S2); (S3); (S14); (S18); (S24); (S4,S12,S28); other populations	0.86346***	-0.02364***	0.86023***
8	(S3); (S4); (S18); (S22); (S24); (S33); (S12,S28); other populations	0.8529***	-0.02776***	0.84882***
9	(S2); (S14); (S16); (S18); (S22); (S24); (S33); (S4,S12,S28); other populations	0.83429***	-0.03371***	0.8287***
10	(S2); (S3); (S4); (S14); (S18); (S24); (S29); (S30); (S12,S28); other populations	0.84056***	-0.01981***	0.8374***

Statistical significance is indicated by stars (* *P* < 0.05, ** *P* < 0.01, *** *P* < 0.001).

**Fig 3:**
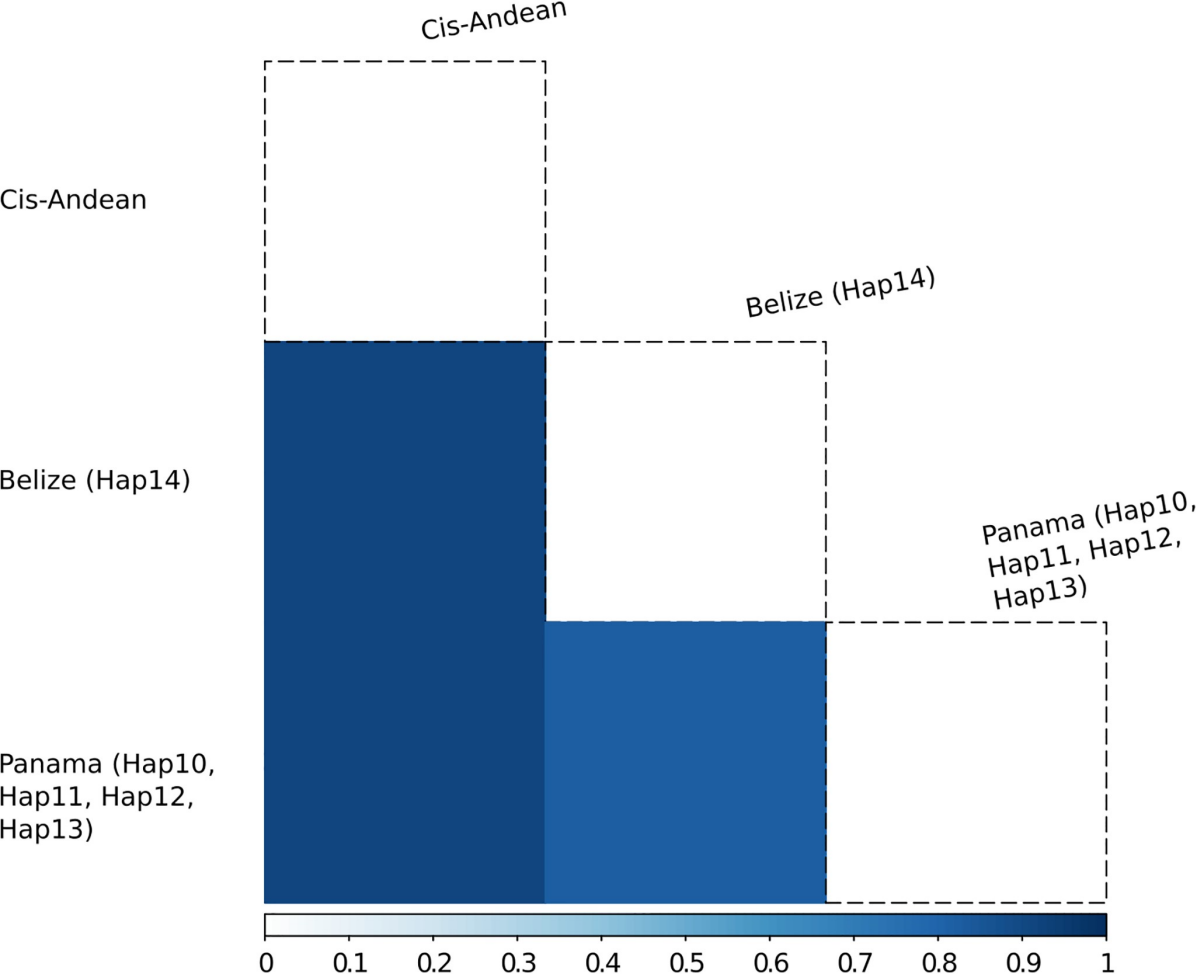
Population pairwise differences (Φ_ST_) among the three clusters suggested by SAMOVA for *R*. *naso*. The color scale refers to the gradient of Φ_ST_ values, from no difference (Φ_ST_ = 0) to complete difference (Φ_ST_ = 1).

The general genetic structure among populations of *R*. *naso* (grouped according to the SAMOVA test) was highly significant (Φ_ST_ = 0.91, *P* < 0.001), while the pairwise Φ_ST_ values among the three major clusters varied from 0.83 to 0.92 (Fig 3, S3 Table). The cis-Andean populations were the most divergent, first compared to those of Belize (Hap14) (Φ_ST_ = 0.92, *P* < 0.001), then compared to those of Panama (Hap10, Hap11, Hap12 and Hap13) (Φ_ST_ = 0.91, *P* < 0.001) (Fig 3, S3 Table). In general, for the populations analyzed, a moderate correlation between genetic distance and geographic distance was detected, and was significant (*r* = 0.55, *P* = 0.00).

The *COI* distances recovered with the Kimura two-parameter (K2P) model among different species of Emballonuridae varied from 0.071 to 0.300. The mean distance within the *R*. *naso* species was 0.030, while the divergence between the cis-Andean and trans-Andean lineages was 0.110. On the other hand, the divergences recovered with *p*-distance varied from 0.066 to 0.244 among different species of the Emballonuridae family, whereas the distance between cis-Andean and trans-Andean lineages of *R*. *naso* was 0.100 (S4–S6 Tables).

### Morphometric analysis

**Fig 4:**
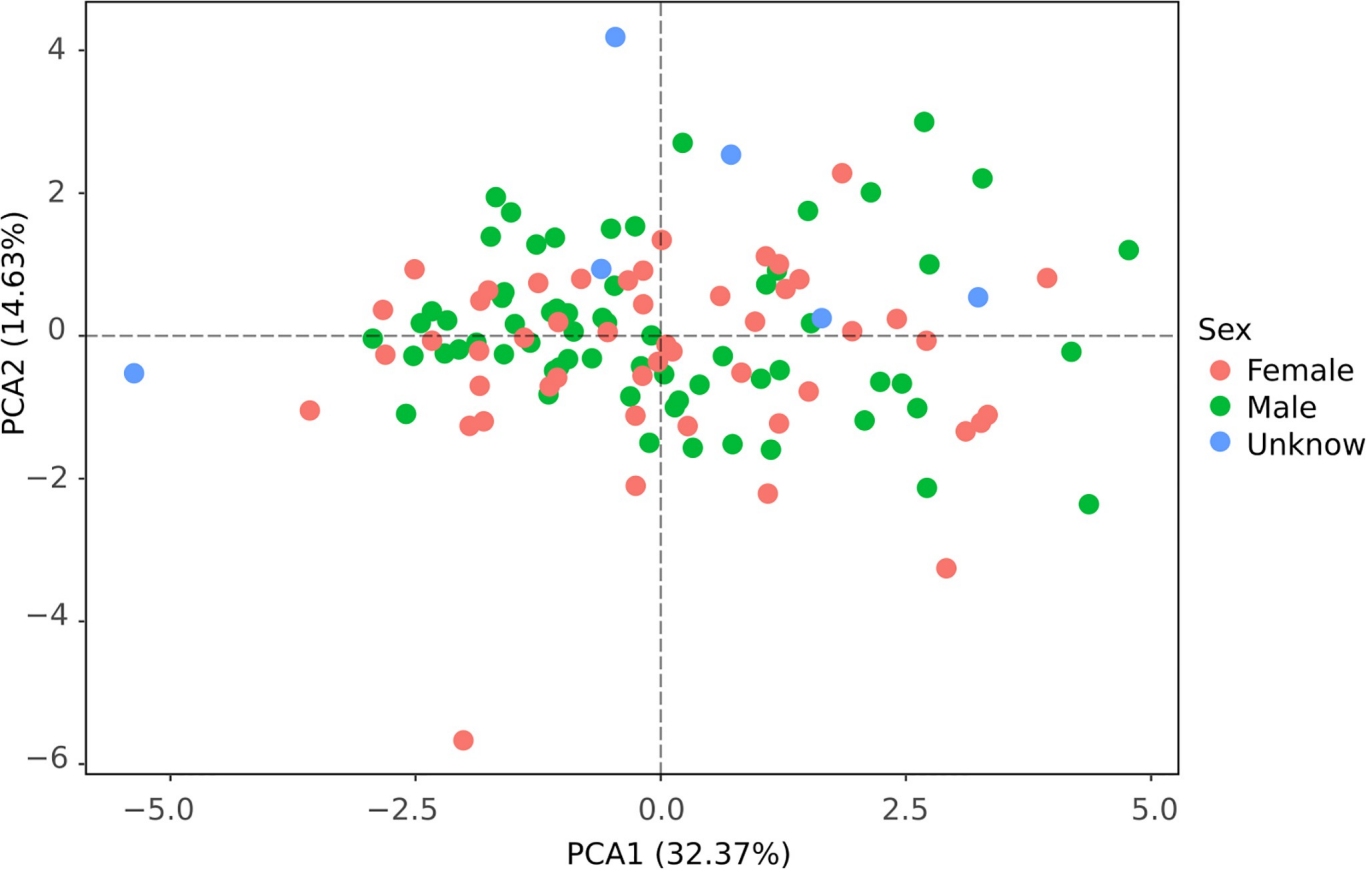
Principal Component Analysis performed with one external (forearm) and 10 craniodental measurements obtained for 121 specimens of *Rhynchonycteris naso* comparing the sexes. Some specimens did not have information about the sex, and were identified as “unknown”.

**Fig 5:**
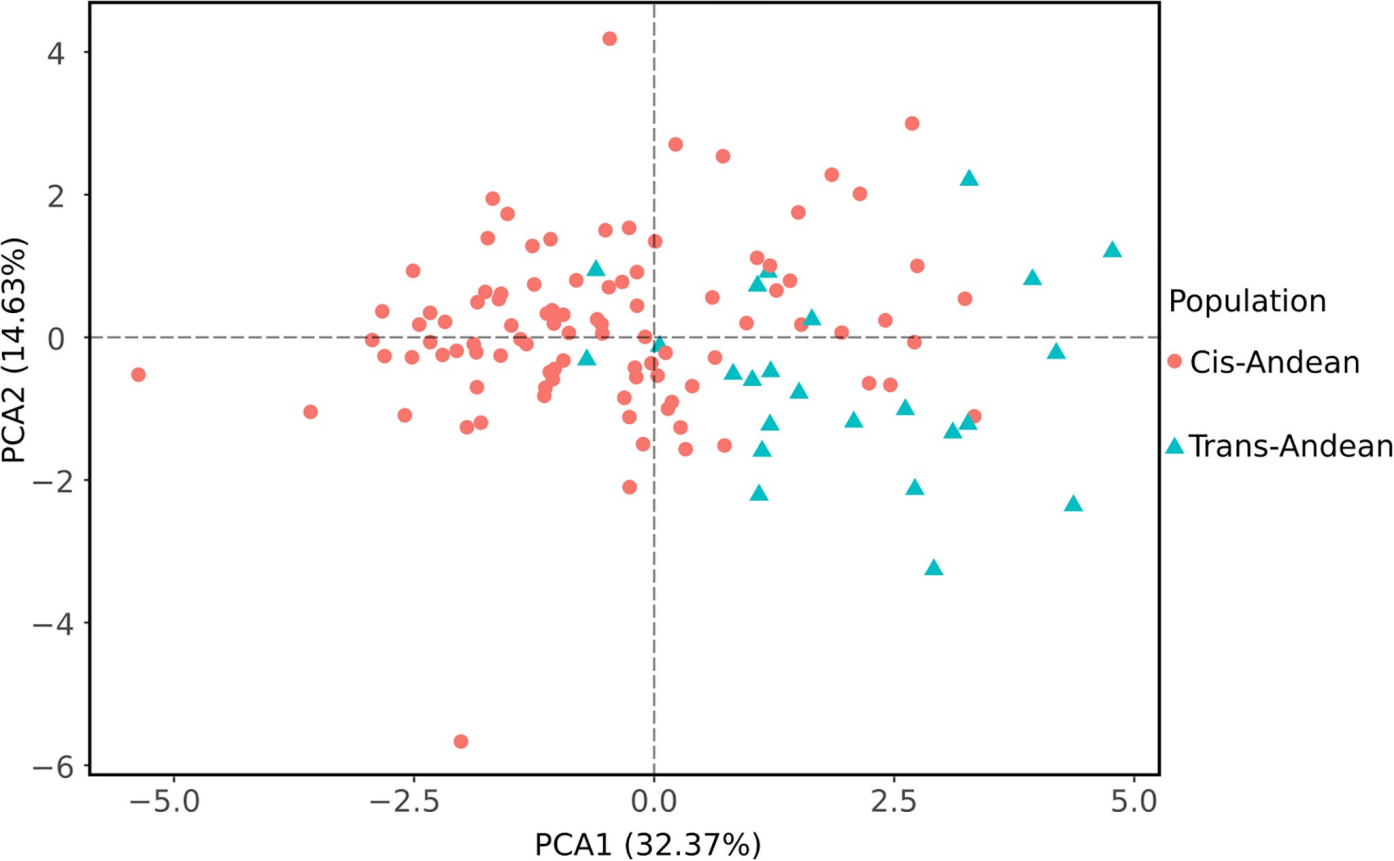
Principal Component Analysis performed with one external (forearm) and 10 craniodental measurements obtained for 121 specimens of *Rhynchonycteris naso* comparing cis-Andean and trans-Andean populations.

When comparing sexes, the PCA did not show any difference in the morphospace (Fig 4). On the other hand, there were apparent differences between cis-Andean and trans-Andean populations in the morphospace, specially regarding PCA1, which explained 32% of the total variation (Fig 5).

**Fig 6:**
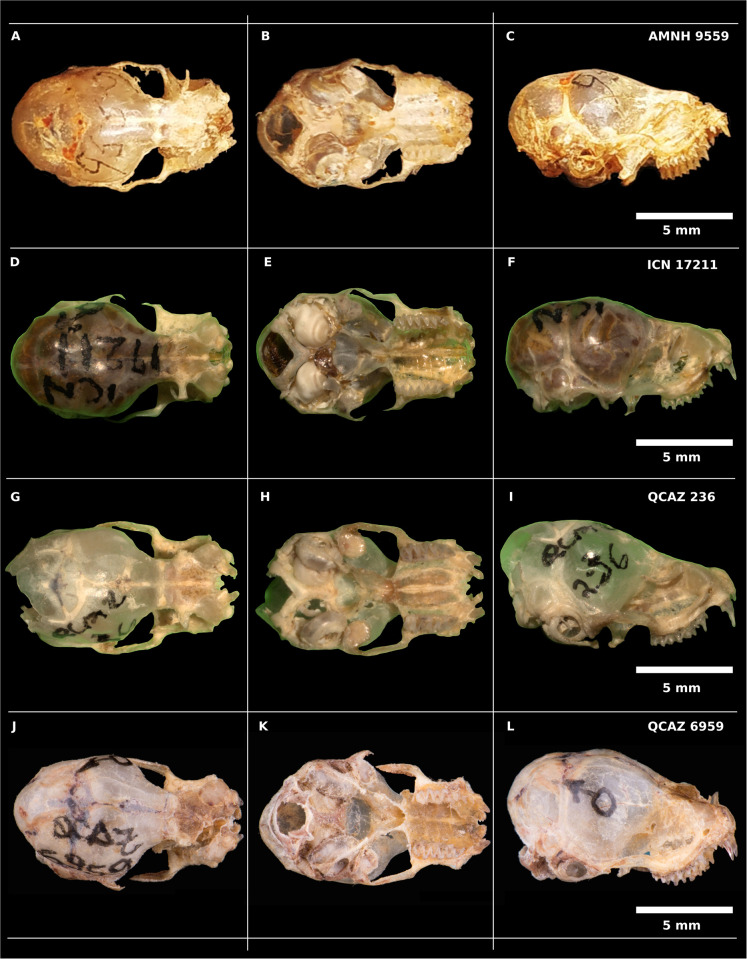
Dorsal, ventral and lateral view of the skulls of *R*. *naso*. (A-C) sex not determined, from Costa Rica (trans-Andean). (D-F) male from Colombia (trans-Andean). (G-I) male from Ecuador (trans-Andean). (J-L) male from Ecuador (cis-Andean). Photo authors (J-L): Andrea Caicedo y Cristian Poveda.

Regarding skull morphology, we were able to identify two general morphotypes without a particular geographic pattern (Fig 6). Morphotype I (Fig 6A–6F and 6J–6L) is characterized by the union of the upper incisors by the palate region (absence of diastema); in ventral view, the maxillary bone protrudes laterally to the molars. Morphotype II (Fig 6G–6I) presents a diastema between the upper incisors in the palate region; in ventral view, the maxillary bone does not protrude laterally to the molars; in lateral view, it seems that the anterior part of the skull (maxilla and nasal region) is narrower when compared with morphotype I. Also, the skull of morphotype II has a more pronounced curvature in the transition between the frontal bone and the maxilla when compared with morphotype I.

Although the PCA did not show differences between sexes in the morphospace, the PERMANOVA analysis indicated that there are significant differences between them (*P* < 0.001). The same happened when cis-Andean and trans-Andean populations were compared. In general, trans-Andean populations are smaller than cis-Andean populations (Table 2). For skull length, the mean of cis-Andean populations were higher (males, 11.6 mm, *n* = 50; females, 11.6 mm, *n* = 40) than those from trans-Andean populations (males, 11.4 mm, *n* = 11; females, 11.2 mm, *n* = 9). The same happened with the forearm length, with the mean of cis-Andean populations higher (males, 37.9 mm, *n* = 48; females, 38.4 mm, *n* = 37) than those of trans-Andean (males, 35.0 mm, *n* = 10; females, 37.3 mm, *n* = 9). Therefore, it can be assumed that a clear differentiation in skull and wing size distinguishes cis-Andean from trans-Andean populations of *R*. *naso*.

**Table 2:** Craniodental and forearm measurements (mm) obtained for different specimens of *Rhynchonycteris naso* according to their sex and general location. Descriptive statistics are presented as mean (minimum–maximum) standard deviation [sample size].

	*Rhynchonycteris naso* (cis-Andean)		*Rhynchonycteris naso* (trans-Andean)	
	Female	Male	Female	Male
**GSL**	11.6 (10.9–12.1) 0.3 [40]	11.6 (10.9–12.2) 0.3 [50]	11.2 (10.7–11.6) 0.3 [9]	11.4 (11.0–11.8) 0.3 [11]
**CBL**	10.7 (9.9–11.3) 0.3 [38]	10.6 (9.9–11.2) 0.3 [50]	10.3 (9.9–10.6) 0.2 [9]	10.3 (9.8–10.7) 0.3 [11]
**UP_CANIN**	1.3 (1.0–1.5) 0.1 [40]	1.3 (1.0–1.7) 0.2 [50]	1.3 (1.0–1.8) 0.2 [9]	1.3 (1.1–1.8) 0.2 [13]
**BR_BCASE**	6.0 (5.4–6.7) 0.3 [39]	6.0 (5.7–6.5) 0.2 [51]	5.9 (5.7–6.2) 0.2 [9]	5.8 (5.6–6.1) 0.2 [13]
**MASTOID**	6.3 (5.2–6.7) 0.3 [38]	6.3 (5.6–6.7) 0.3 [50]	6.1 (5.4–6.5) 0.3 [9]	6.1 (5.8–6.3) 0.2 [12]
**TR_SUP**	4.3 (3.9–4.5) 0.1 [40]	4.3 (4.0–4.6) 0.1 [51]	4.1 (3.9–4.3) 0.1 [9]	4.1 (4.0–4.3) 0.1 [13]
**M3-M3**	2.7 (2.2–3.6) 0.2 [39]	2.7 (1.9–3.0) 0.2 [44]	2.8 (2.5–3.1) 0.2 [8]	2.6 (2.2–3.0) 0.2 [12]
**C1-C1**	2.2 (1.9–2.6) 0.2 [40]	2.4 (2.0–4.1) 0.3 [45]	2.2 (1.9–2.6) 0.2 [9]	2.3 (1.7–2.9) 0.3 [12]
**DENT_LEN**	8.0 (7.3–8.8) 0.4 [36]	8.0 (7.2–8.6) 0.3 [46]	7.7 (7.0–8.2) 0.4 [7]	7.7 (7.1–8.1) 0.4 [10]
**TR_INF**	4.4 (3.1–4.7) 0.3 [37]	4.5 (4.3–5.0) 0.1 [46]	4.2 (3.9–4.5) 0.2 [7]	4.3 (4.1–4.6) 0.1 [11]
**FA**	38.4 (32.6–41.6) 1.9 [37]	37.9 (31.8–40.1) 1.5 [48]	37.3 (35.6–39.5) 1.3 [9]	35.0 (31.1–37.4) 2.1 [10]

### Ecological niche modeling

**Fig 7:**
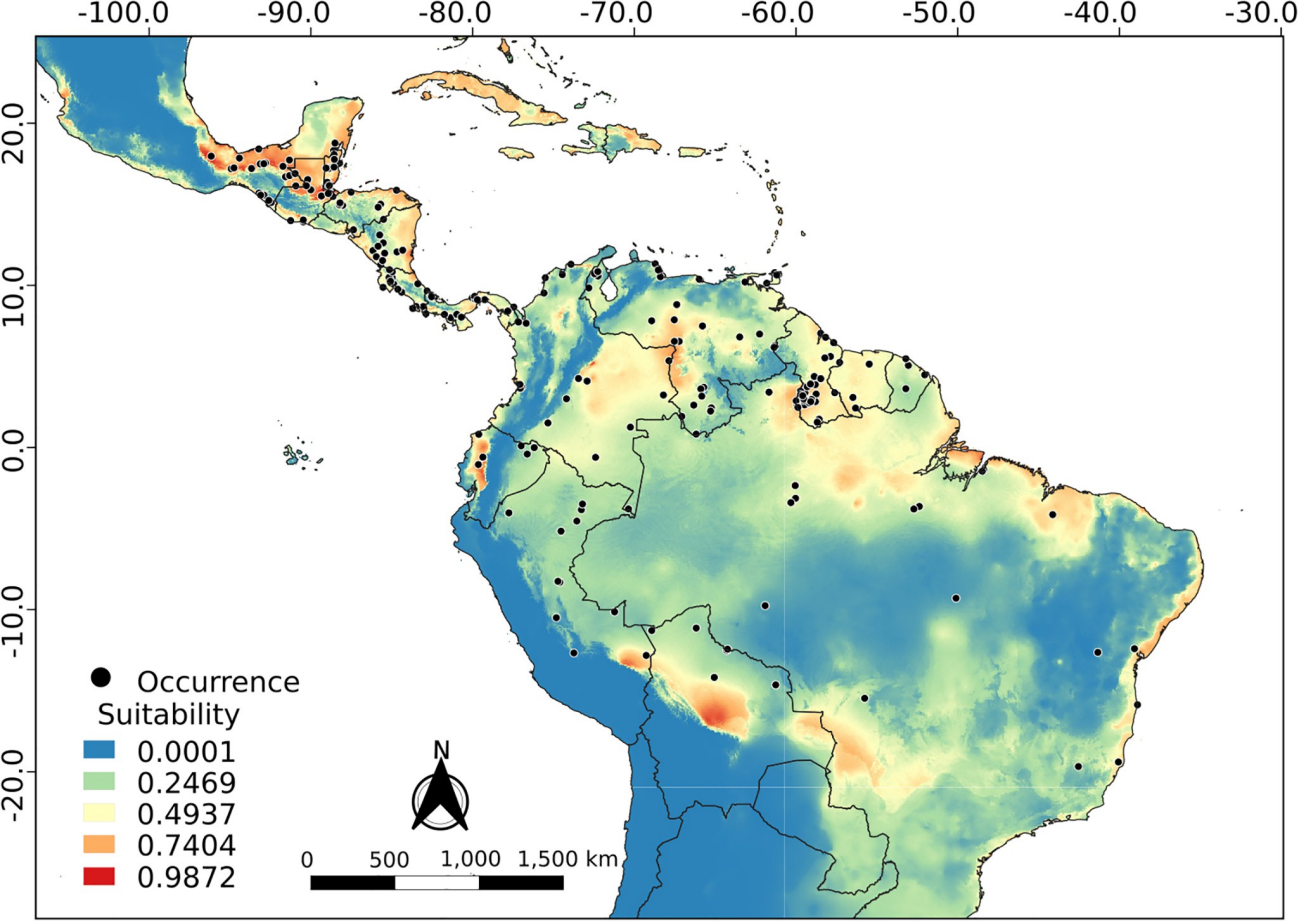
Projection of ecological niche modeling under current conditions for *R*. *naso* considered as a whole.

The best ENM configurations obtained for *R*. *naso* as a whole or for each of its clusters are presented in Table 3. The projection of the total model for current climatic conditions (Fig 7 and S1 Fig) shows a high suitability in coastal regions from south of Mexico to Northeast Brazil, the Amazon region, the Llanos region in Colombia and Venezuela and a patch covering the central-North of Bolivia and the southeast of Peru. Furthermore, ENM analysis suggests that the Andean Cordillera and the Sistema Coriano in Venezuela (mountain system, transition between the Cordillera de la Costa and the Andes Mountains) could act as a climatic barrier (suitability = 0) between cis-Andean and trans-Andean populations. Also, according to this model, part of the Brazilian Caatinga and Cerrado biomes would act as a climatic barrier, isolating populations of *R*. *naso* in the forests of the east coast of the country.

**Table 3:** Performance metrics obtained for ENM reconstructed for *R*. *naso* as a whole or for each of its clusters using different parameter settings regarding regularization multiplier (RMs), feature classes (FCs), and sets of enviromental data.

Species (group)	RMs	FCs	Set	Partial ROC	Omission rate 5%	AICc	Delta AICc	Number of parameters
***R*. *naso* (Total)**	4	LPTH	1	0	0.02	5672.35	0	23
***R*. *naso* (Total)**	1	PTH	3	0	0.03	5673.14	0.79	51
***R*. *naso* (cis-Andean)**	4	LPTH	1	0	0	2920.86	0	20
***R*. *naso* (cis-Andean)**	5	LPTH	1	0	0	2921.86	1	16
***R*. *naso* (trans-Andean)**	4	LQP	1	0	0	2336.94	0	10

The FCs are as follows: Linear = L, quadratic = Q, product = P, threshold = T and hinge = H.

**Fig 8:**
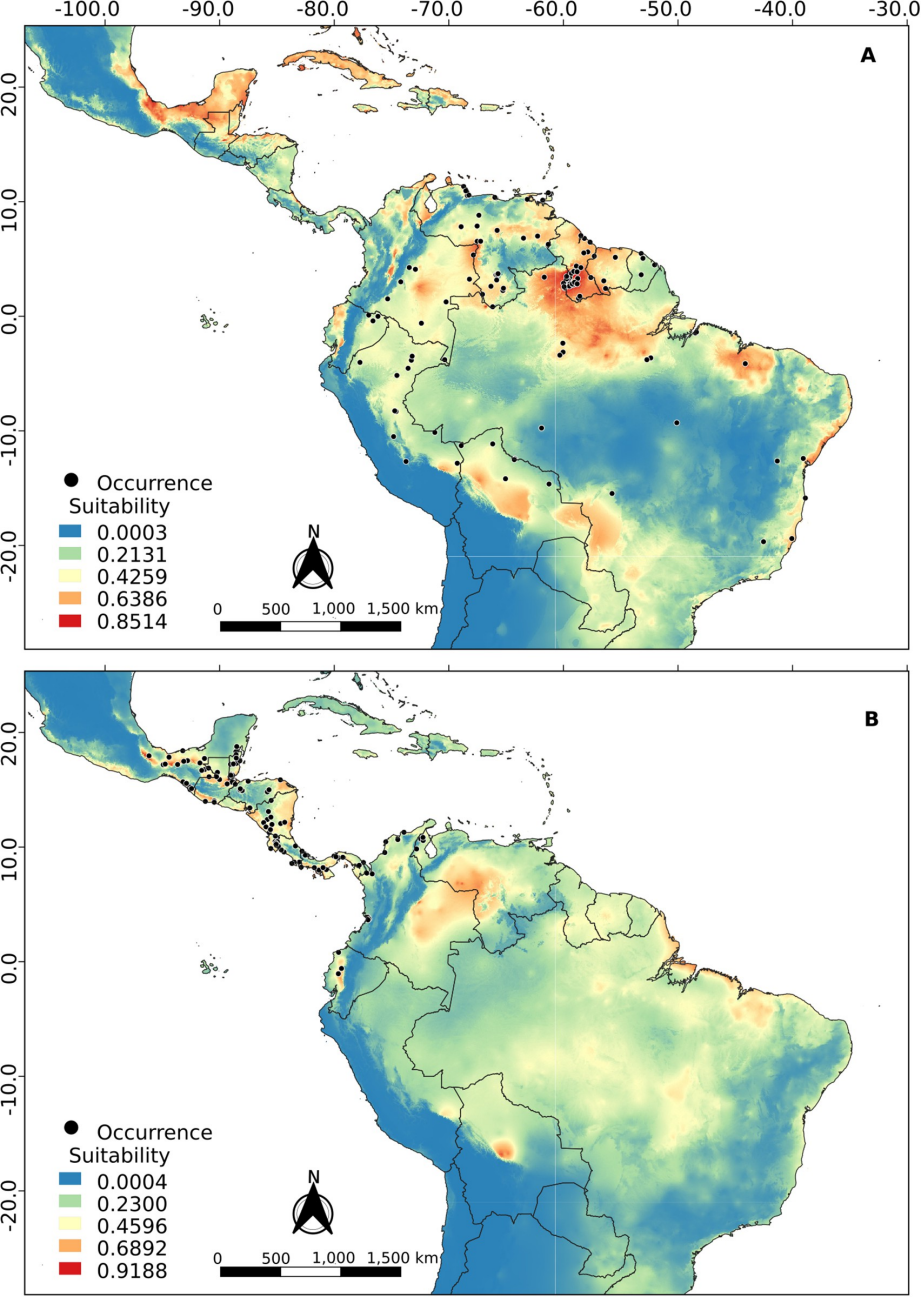
Projection of ecological niche modeling under current conditions for the (A) cis-Andean and (B) trans-Andean clusters of *R*. *naso*.

The cis-Andean and trans-Andean models showed a similar result compared to the total model. For the cis-Andean model (Fig 8A and S1 Fig), the Andean Cordillera seems to be acting as a climatic barrier, but the Sistema Coriano and northeast of Caatinga biome do not seem to be a problem. The trans-Andean model (Fig 8B and S1 Fig) is similar to the cis-Andean one, but the particularity of this one is that it presents the Yaracuy depression (a geographic feature located in northwest Venezuela) as a putative connection between the two populations.Regarding the importance presented by each variable to the models, precipitation of driest quarter (40.6%) and annual precipitation (13.8%) were the most influential to the cis-Andean population. On the other hand, seasonal temperature (20.8%) and precipitation of the wettest month (17.6%) were the most important in the trans-Andean population.

**Fig 9:**
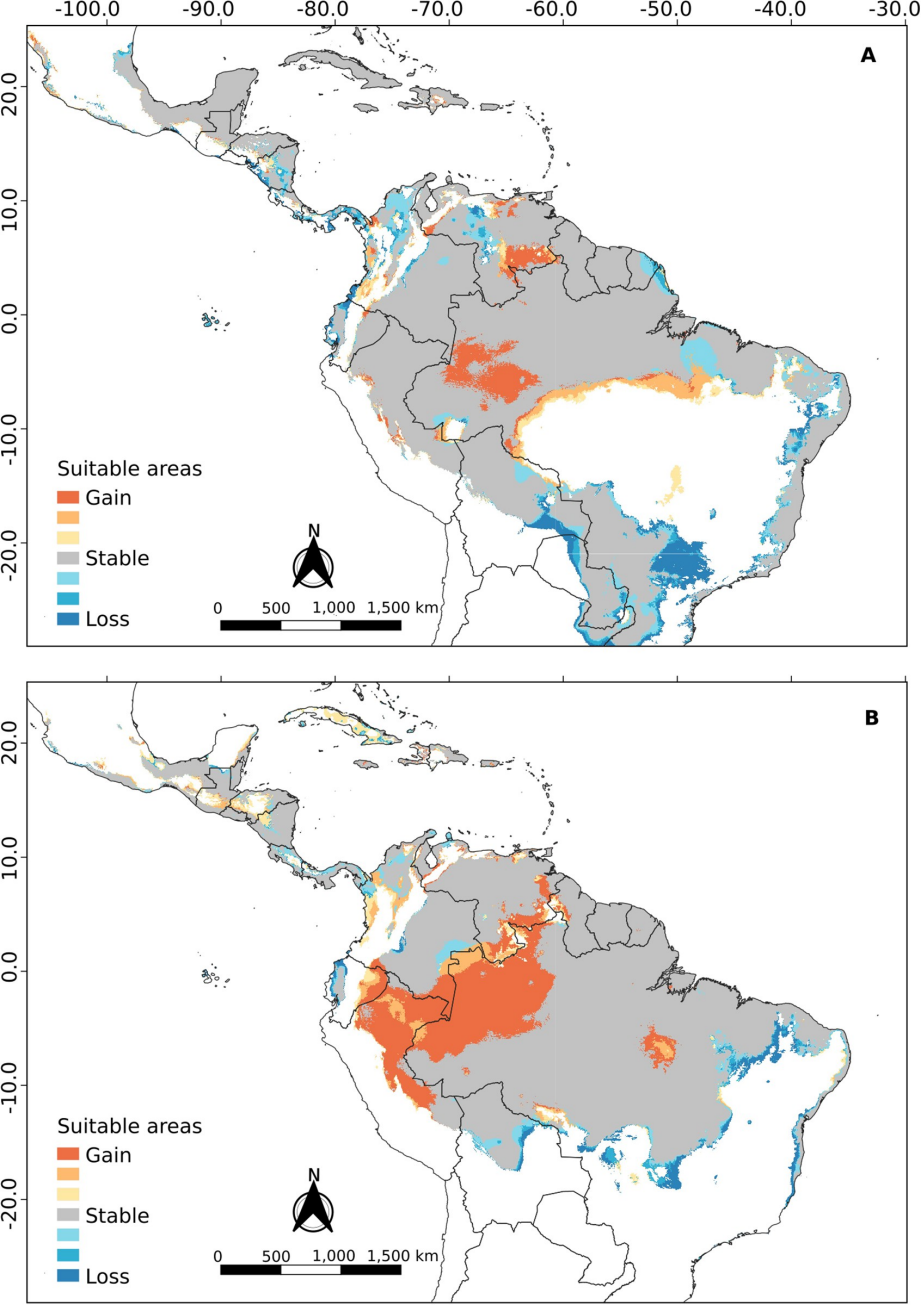
Projection of ecological niche modeling in the middle Holocene for the (A) cis-Andean and (B) trans-Andean clusters of *R*. *naso*.

In past climate model projections, the mid-Holocene map (Fig 9) showed that cis-Andean populations would have an increase in climatically suitable areas in the Andes region of Venezuela. It is important to note that the increase in this area would allow the Táchira depression (a geographic feature in the east of the Andes, separating the Tamá massif to the west and the Sierra de Mérida to the east) in Venezuela to act as a new passage to cis-Andean populations across the Cordillera. Also, there would be a reduction in some areas of southern and eastern Brazil. On the other hand, trans-Andean populations would have an increase in climatically suitable areas in the Andes region in Venezuela, but this increase does not suggest the Táchira depression region as a climatically suitable region for the passage of trans-Andean populations through the mountain range. One of the general circulation models (GCM) shows the loss of climatically suitable areas in the Sistema Coriano in the northwest of Venezuela, which would leave the trans-Andean populations isolated from the cis-Andean populations in the middle Holocene.

**Fig 10:**
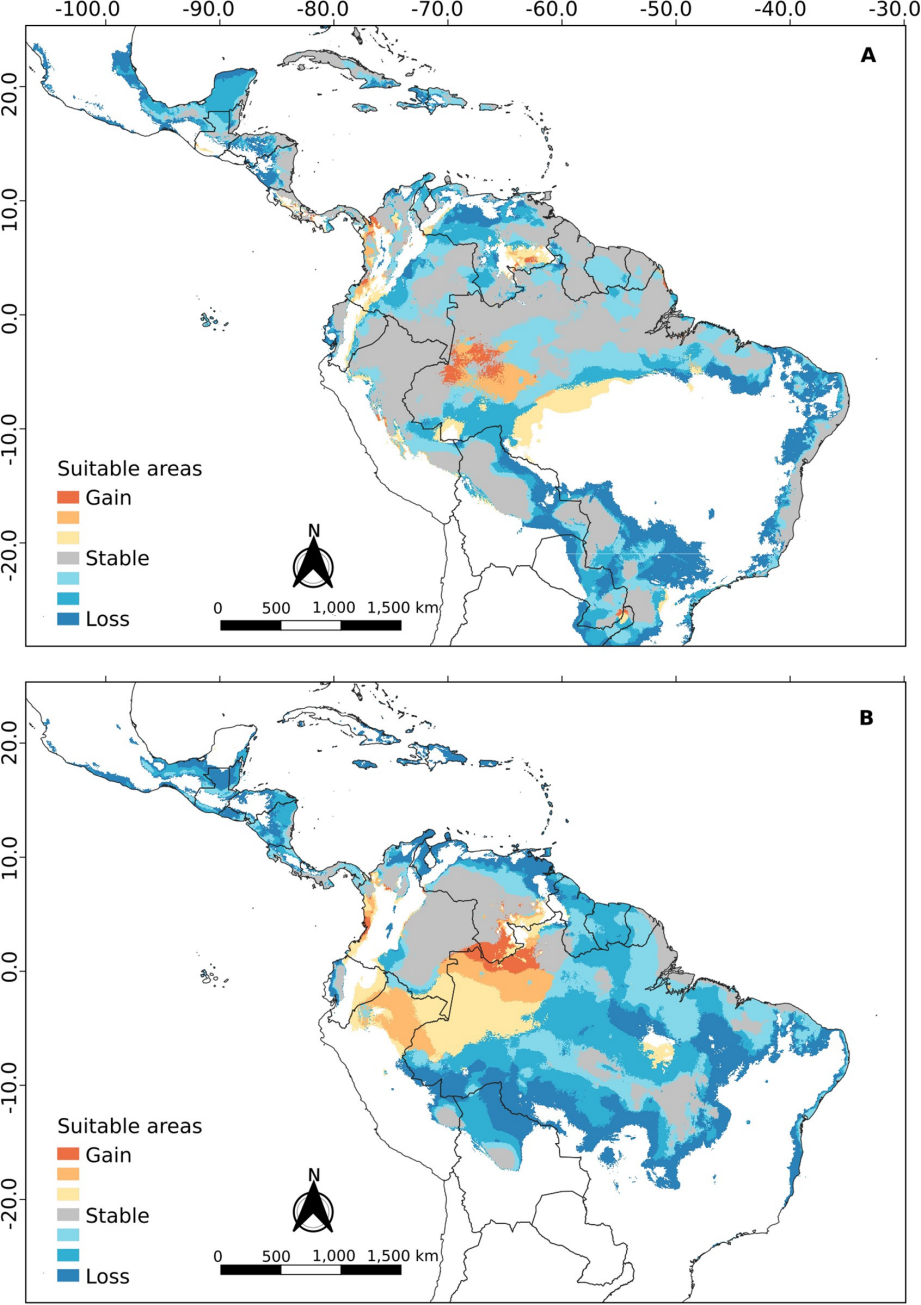
Projection of ecological niche modeling in the last glacial maximum for the (A) cis-Andean and (B) trans-Andean clusters of of *R*. *naso*.

At the last glacial maximum (Fig 10), climatically suitable areas for cis-Andean populations are reduced, especially in the Sistema Coriano, Los Llanos from Venezuela and Colombia, and the Andes region from Venezuela to Peru. In addition to this reduction of areas, the populations of the east coast of Brazil (Rio Grande do Norte, to Rio do Janeiro) would be separated from those encountered in the rest of Brazil by the loss of suitable areas in the northeast region of the country (Maranhão, to Ceará, and part of Rio Grande do Norte). Trans-Andean populations would have a considerable reduction in climatically suitable areas, limiting their populations to the coasts of the Caribbean Sea in Nicaragua, in southern Costa Rica, on the coasts of the Pacific Ocean in Panama, and in northwest Colombia.

**Fig 11:**
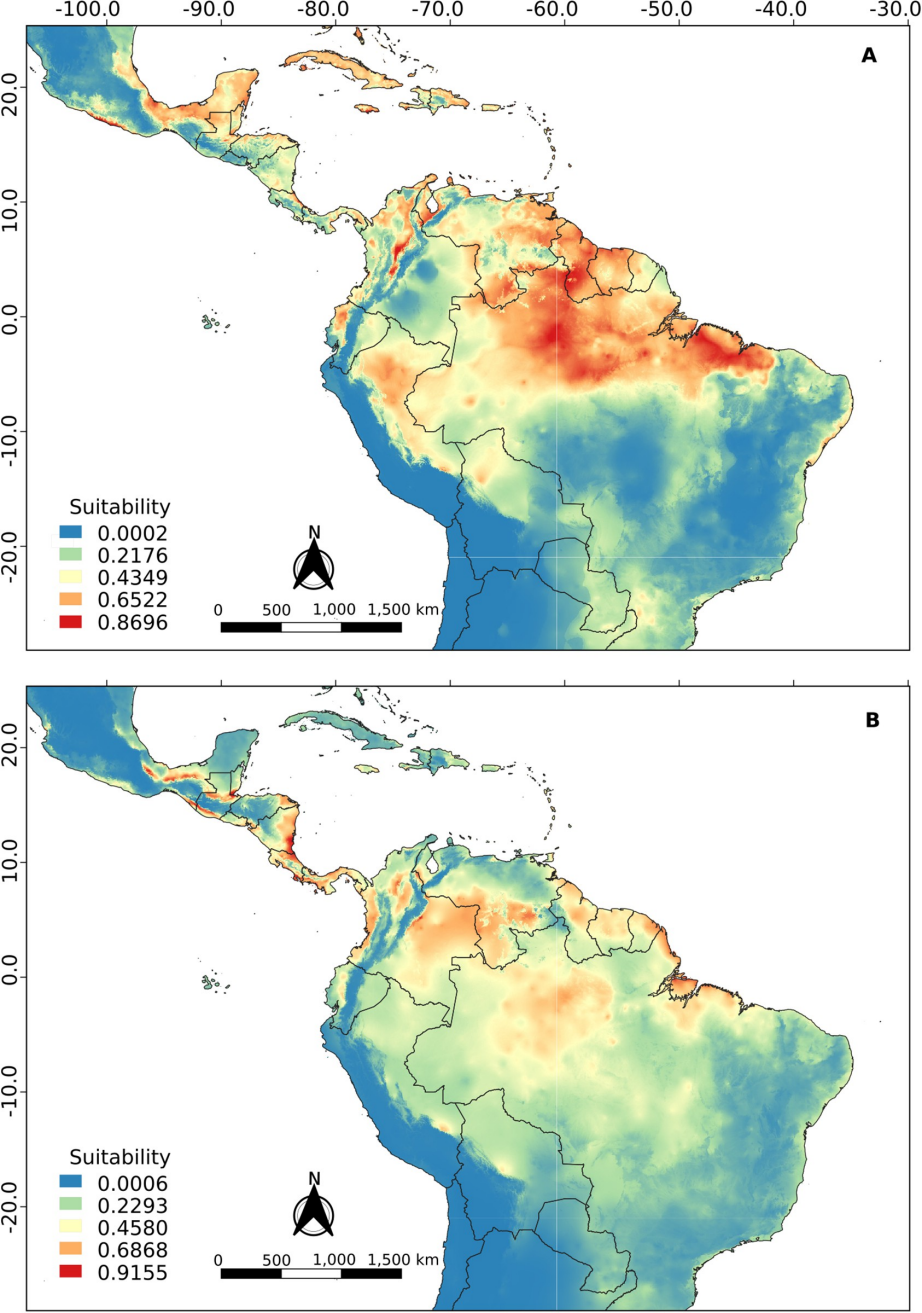
Projection of ecological niche modeling in the last Interglacial for the (A) cis-Andean and (B) trans-Andean clusters of *R*. *naso*.

In the last Interglacial (Fig 11) the models showed a distribution of climatically suitable areas that is similar to those found in the present projections for both populations.

## Discussion

Integrative approaches are being commonly helping to overcome the taxonomic impediment, leading to an acceleration on the delimitation and on the discovery of new species [62–64]. This study adds to this scenario in supporting the presence of cryptic or unrecognized diversity within the morphotope previously assigned to *Rhynchonycteris naso*. In fact, molecular analysis suggested a deep subdivision of this species in two different clusters, that seem to yield incipient morphological and ecological differences. ENM strategies further reinforced this outcome, by presenting a climatic barrier that extends along the Andean Cordillera and the Sistema Coriano in Venezuela that could have resulted in the divergence between cis-Andean and trans-Andean (Belize and Panama) populations of *R*. *naso*.

The genetic divergence found here between trans and cis-Andean populations for the *COI* gene was 11%, which is above the mean of 7.8% of genetic difference found by Clare *et al*. [65] for the same marker when they compared 47 genera of bats. Clare *et al*. [65] and Clare *et al*. [66] also analyzed the mean *COI* intraspecific divergence of 87 species of bats belonging to seven families in Guyana, and 163 species of bats belonging to nine Neotropical families, respectively, and found mean intraspecific values of 0.6% and 1.38%, which are quite lower than the 3% found here for *R*. *naso*. On the other hand, Martins *et al*. [67] found a genetic diversity range of 6–11% for *Desmodus rotundus* when they analyzed the mitochondrial *Cytb* gene. The authors state that these values are among the highest ones that have already been described for Neotropical bats. Although Martins *et al*. [67] used a mitochondrial gene different from that used in our work, which is likely to have a distinct evolution rate, Tobe *et al*. [68] affirm that the *Cytb* gene is more variable at intra and interspecific levels when compared to the *COI* gene. This reinforces the idea that the genetic distance of 11% found in the populations of *R*. *naso* suggest the presence of cryptic species. This is a very common occurrence in bats, and is usually associated with the low number of taxonomic works, which are needed to clarify the status of species [65,69,70].

Another source of evidence supporting subdivision of *R*. *naso* in two clusters is provided by the Network analysis, where each of them encompasses a distinct haplogroup, differing by at least 47 substitutions. This is also quite above the results previously presented for different species by other authors. Baird *et al*. [71], for example, studied the relationships between two species of Vespertilionidae bats, *Aeoreste cinereus* and *Aeorestes semotus*, of North America and Hawaii, and found a difference of at least 13 substitutions using the *COI* gene, and genetic divergences of 4.2% using the *Cytb* gene. On the other hand, Demos *et al*. [72] studied the genetic diversity of particular clades of Nycteridae bats using the *Cytb* gene, and detected that populations of *Nycteris thebaica* clade 1 and *Nycteris thebaica* clade 3 differ by approximately 40 substitutions, and 5% of genetic divergence. Thus, the differences found between the two clusters (cis-Andean and trans-Andean) of *R*. *naso* provide significant support to their diversification and isolation, suggesting the species could be subdivided in at least two species.

Although PCA analyzes (Fig 4) showed that males and females of *R*. *naso* are homogeneous within morphospace, it recovered a distinct pattern when cis-Andean and trans-Andean populations are compared. Besides, trans-Andean populations revealed smaller than cis-Andean populations. The visual analysis of the morphology of the skull reveals the presence of two distinct morphotypes without an apparent geographic pattern. This is not surprising, since morphological stasis can be favored by selection during cladogenesis, leading to the emergence of new species without apparent morphological change [73]. Alternatively, the faint morphological difference found in this study can be due to a recent speciation process and the absence of the disruptive selection in morphological characters [23]. Afterall, similar ecological niches can direct the morphological homogeneity [23].

In general, the projections resulting from ecological niche modeling in the present and during the Middle Holocene (Figs 8 and 9) showed low suitability along the entire Andes cordillera, to the exception of regions of high suitability in the Yaracuy depression in Venezuela. These regions may have acted as barriers to gene flow between the trans-Andean and cis-Andean populations, promoting a vicariance between populations occurring in both sides of the Andes. Similar results were obtained by Gutiérrez-Pinto *et al*. [74] using birds of the Parulidae family. According to Plumpton and Jones [8] and Hood and Gardner [10], *R*. *naso* has a specific distribution in lowland tropical areas, rarely reaching a maximum of 900 meters of elevation. According to this information, the Andes cordillera would be one of the most efficient barriers to the dispersion of this species.

Furthermore, projections to the last glacial maximum (Fig 10) showed a significant decrease in climatic suitability regions (compared to those in the present), with restricted areas presenting suitable climatic conditions putatively acting as refuges for populations of *R*. *naso*. These patterns may have influenced the genetic diversification, the speciation and the distribution area of the populations studied here [75]. Thus, glacial and interglacial cycles may have presented important effects on the isolation of populations in warmer and humid areas in Central America (e.g., Panama and Colombia) and South America (e.g., North and West of the Amazon), where individuals could survive during more harsh conditions. Although in the last interglacial the increase in temperatures would have benefitted the occupation of new areas, the Andes cordillera could still have acted as a climatic barrier keeping the two populations apart. Even though, the Sistema Coriano, the Yaracuy depression, and possibly the Tachira depression seem to constitute climatically suitable passages, enabling a putative gene flow between populations that could not be detected here.

## Conclusion

The speciation process is not always accompanied by drastic morphological changes. This commonly makes traditional taxonomy practices to underestimate the real number of biological species currently existing. Nevertheless, especially in the face of the taxonomic impediment and the biodiversity crisis, we emphasize here the importance of integrative approaches to the identification of cryptic species. This task is of utmost importance for the formulation of conservation plans and strategies [73]. In this work we present several evidence demonstrating that there are two clusters within the general morphotype previously assigned to *Rhynchonycteris naso*, and that these probably represent two distinct species. Such recognition is important to understand the real distribution of each taxa, to evaluate their threatens and to promote further biological studies. Only in the face of this new status it will be possible to protect important providers of ecosystem services, as are the insectivorous bats.

## Supporting information

S1 Fig Summary of ENM calibration and projection areas and extrapolation risks for *Rhynchonycteris naso* considered as a whole or for each of its clusters in current conditions. (TIFF)

S2 Fig Majority-rule tree inferred from the combined data set of Embalonurid bats through a partitioned Bayesian analysis. The numbers following the species names correspond to the unique identifiers. Bayesian posterior probabilities are indicated at each node. Branch lengths are proportional to the scale, given in substitutions per nucleotide.
(PDF)

S3 Fig Maximum likelihood individual gene trees, and mitochondrial data combined data set, of Embalonurid bats. Bootstrap probabilities are indicated at each node.
(PDF)

S1 Table List of specimens used for morphological and molecular evaluations. Species with unique identifier number, locality, associated voucher specimen, and GenBank accession number.
(PDF)

S2 Table List of *Rhynchonycteris naso* haplotypes used in this work. The numbers following the species names correspond to the unique identifiers.
(PDF)

S3 Table Pairwise values of genetic differentiation (Φ_ST_) obtained with *COI* sequences among the three clusters of populations of *Rhynchonycteris naso* suggested by SAMOVA analysis. Values below the diagonal are distances. Values above the diagonal are the *p* values.
(PDF)

S4 Table Pairwise values of interspecific genetic distance obtained with *COI* sequences among different species of Embalonurids bats, using the Kimura 2-parameters substitution model. The numbers highlighted in gray refer to the level of sequence divergence within groups.
(PDF)

S5 Table Pairwise values of interspecific genetic distance obtained with *COI* sequences among different species of Embalonurids bats, using *p*-distance. The numbers highlighted in gray refer to the level of sequence divergence within groups.
(PDF)

S6 Table Mean values of intraspecific genetic distance obtained with *COI* sequences for different species of Embalonurids, using kimura 2-parameters substitution model. (PDF)
