# Supplementary figures and images for: Systematics and phylogeography of bats of the genus Rhynchonycteris (Chiroptera: Emballonuridae): Integrating molecular phylogenetics, ecological niche modeling and morphometric data
Source: PLoS One. 2023 May 4;18(5):e0285271. doi: 10.1371/journal.pone.0285271 (PMC10159116; doi:10.1371/journal.pone.0285271)

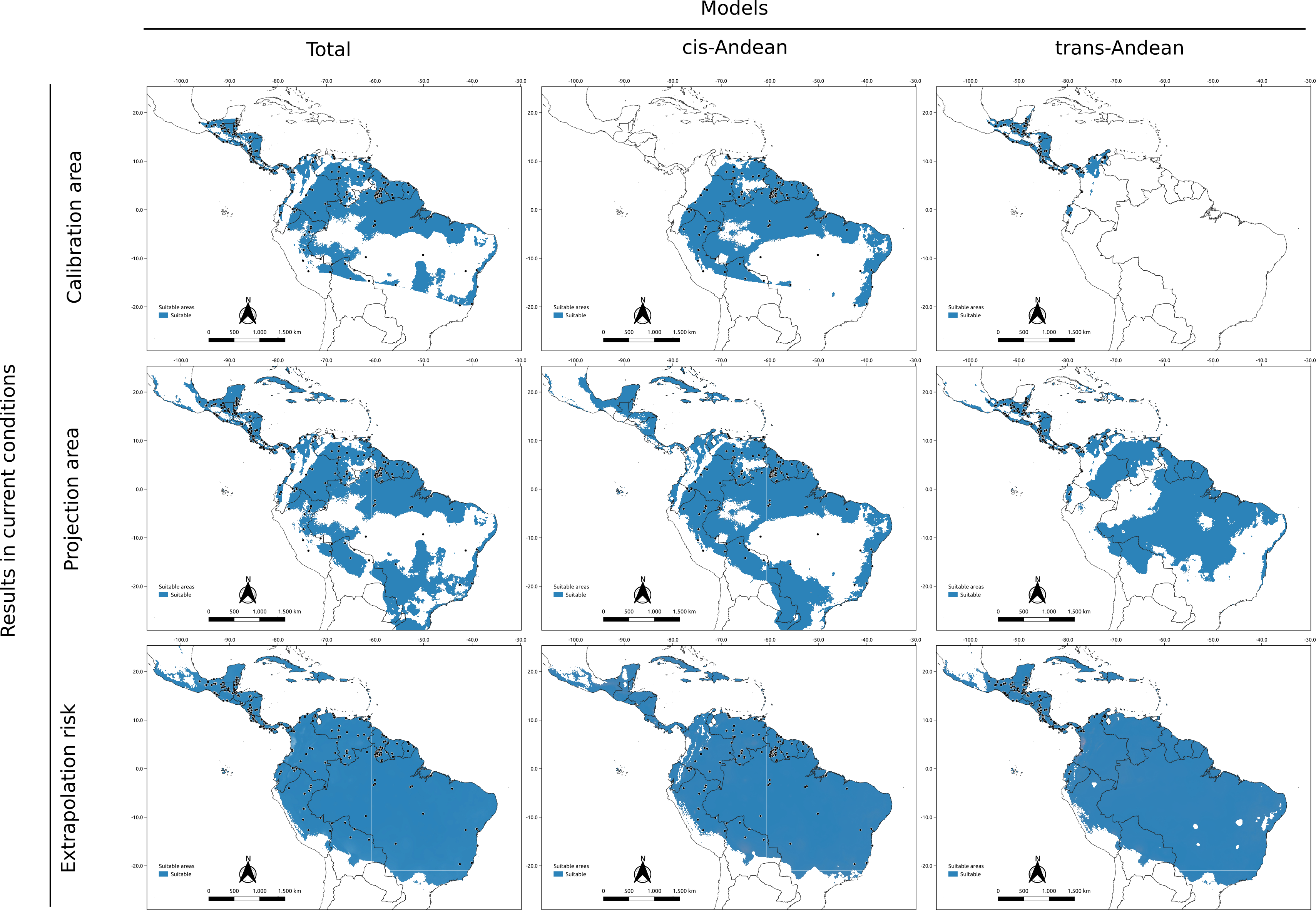

Supplement: S1 Fig — (TIFF) [file pone.0285271.s001.tiff]

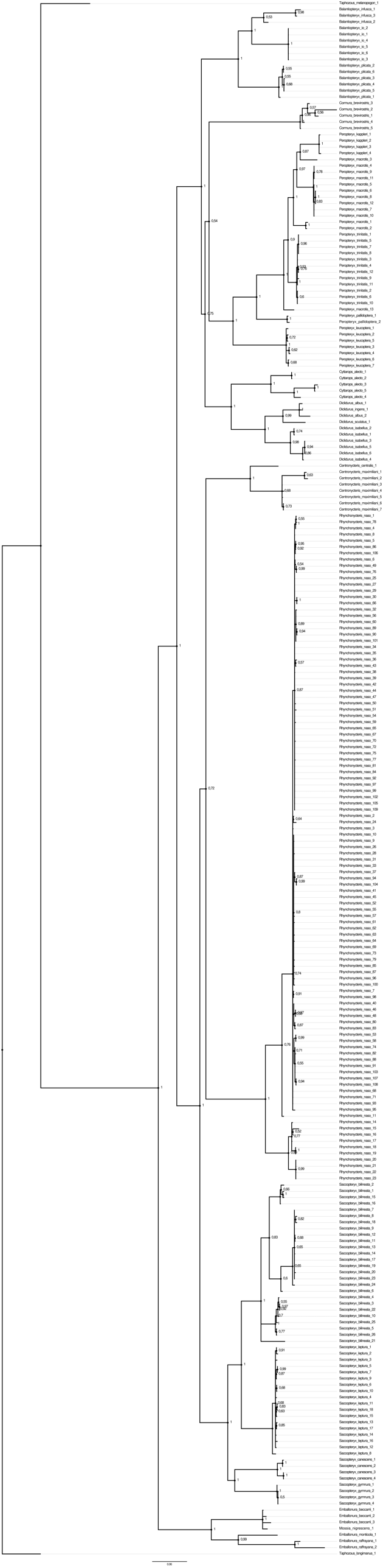

Supplement: S2 Fig — The numbers following the species names correspond to the unique identifiers. Bayesian posterior probabilities are indicated at each node. Branch lengths are proportional to the scale, given in substitutions per nucleotide. (PDF) [file pone.0285271.s002.pdf]
