# Supplementary material for: Systematics and phylogeography of bats of the genus Rhynchonycteris (Chiroptera: Emballonuridae): Integrating molecular phylogenetics, ecological niche modeling and morphometric data
Source: PLoS One. 2023 May 4;18(5):e0285271. doi: 10.1371/journal.pone.0285271 (PMC10159116; doi:10.1371/journal.pone.0285271)

Maximum likelihood *Chd1* gene tree. Bootstrap probabilities are indicated at each node.

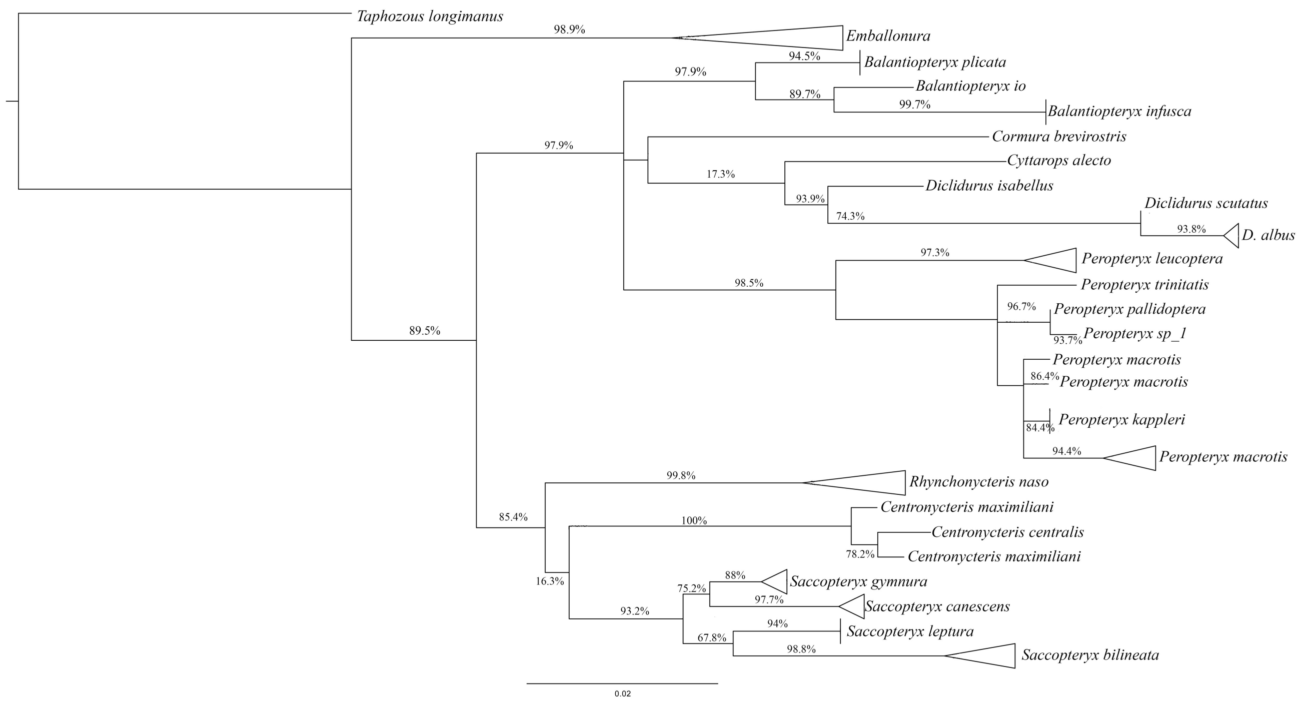

Maximum likelihood *COI* gene tree. Bootstrap probabilities are indicated at each node.

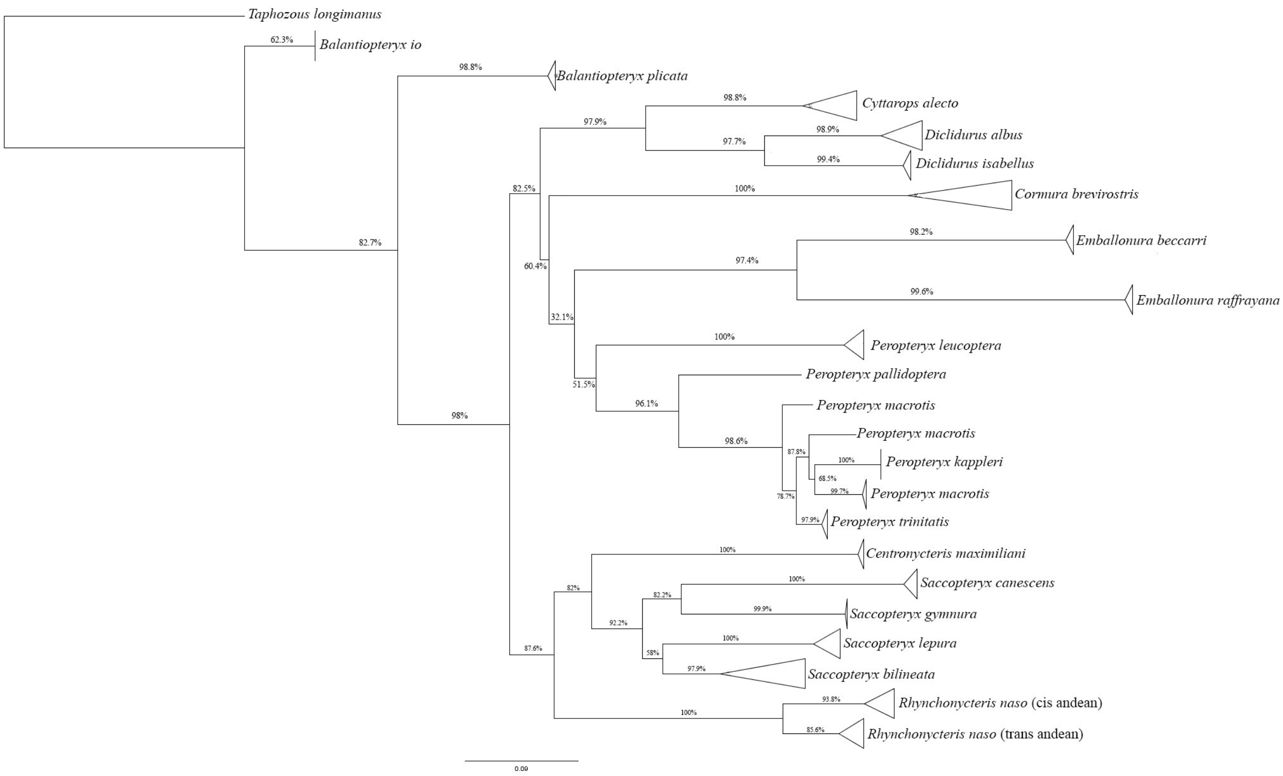

Maximum likelihood *Cytb* gene tree. Bootstrap probabilities are indicated at each node.

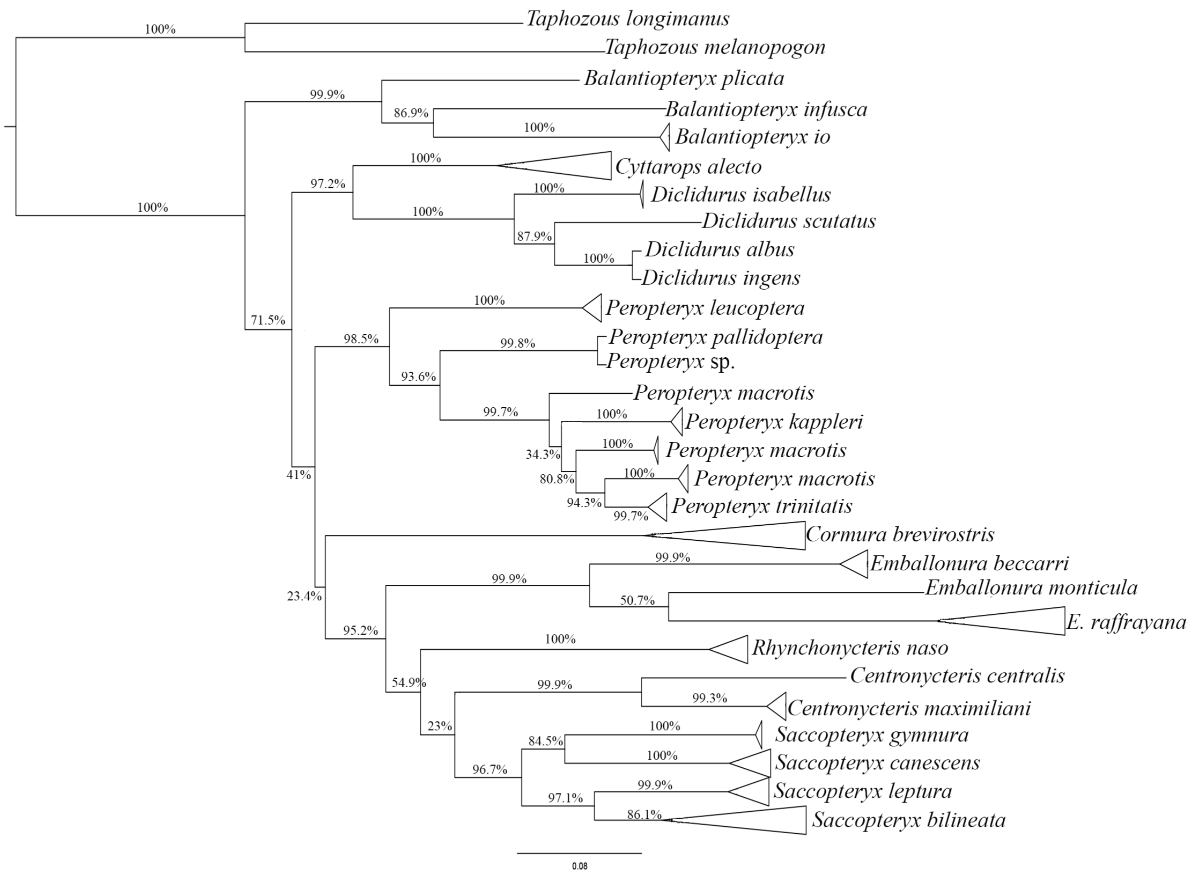

Maximum likelihood *Dby* gene tree. Bootstrap probabilities are indicated at each node.

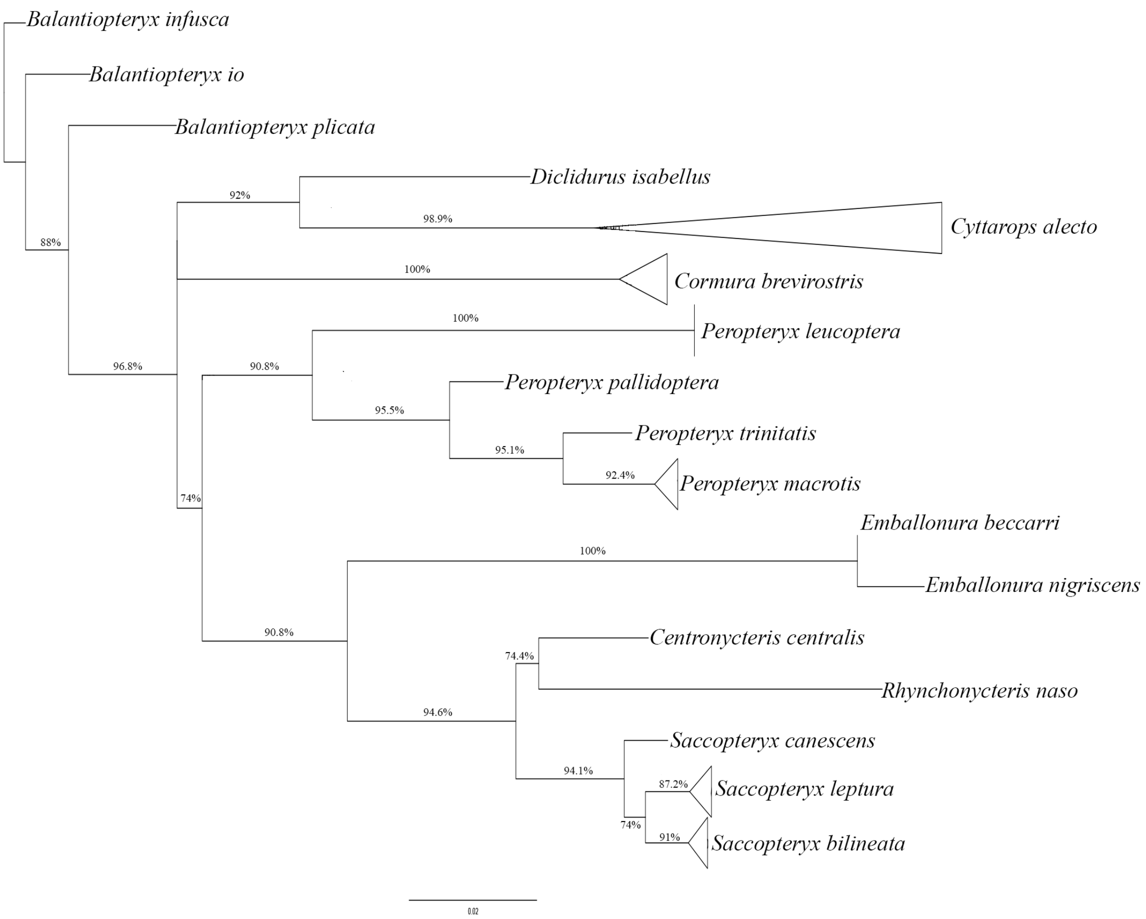

Maximum likelihood *Usp9x* gene tree. Bootstrap probabilities are indicated at each node.

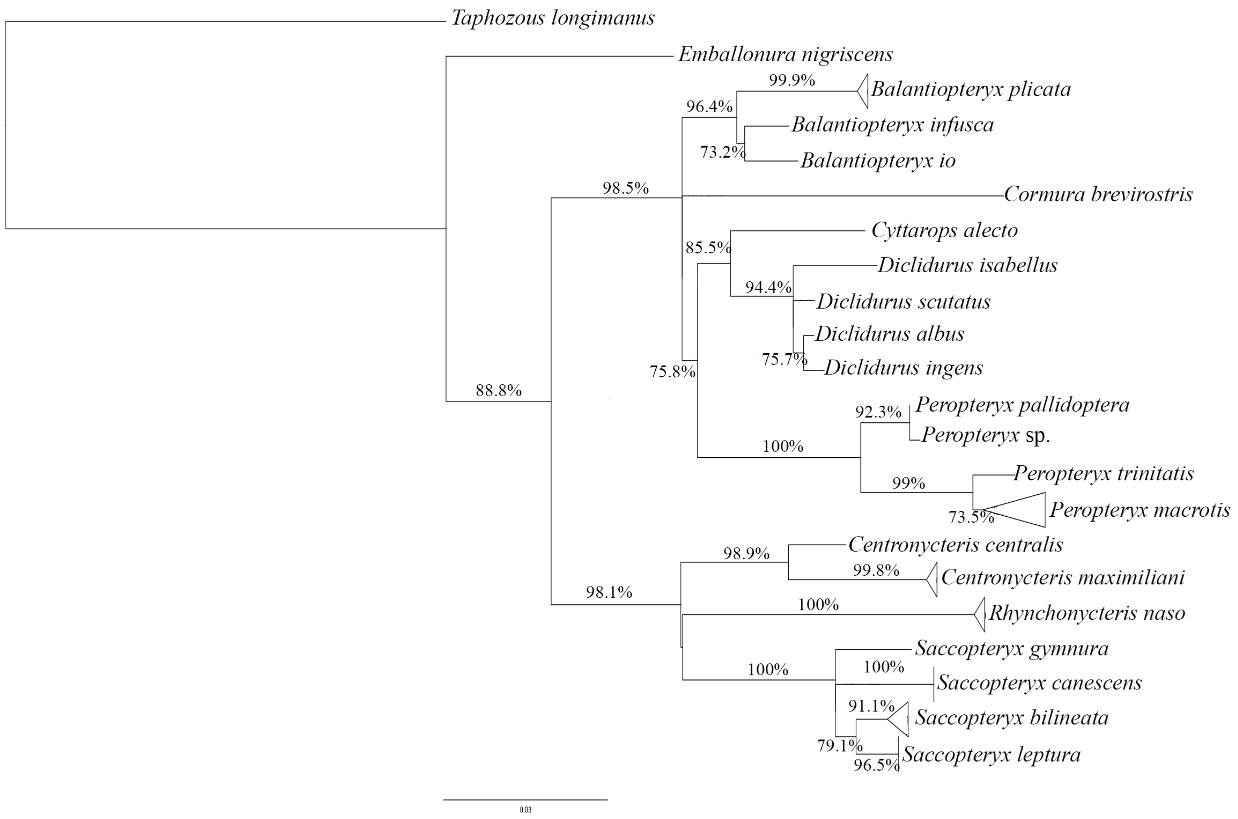

Maximum likelihood mitochondrial matrix gene tree. Bootstrap probabilities are indicated at each node.

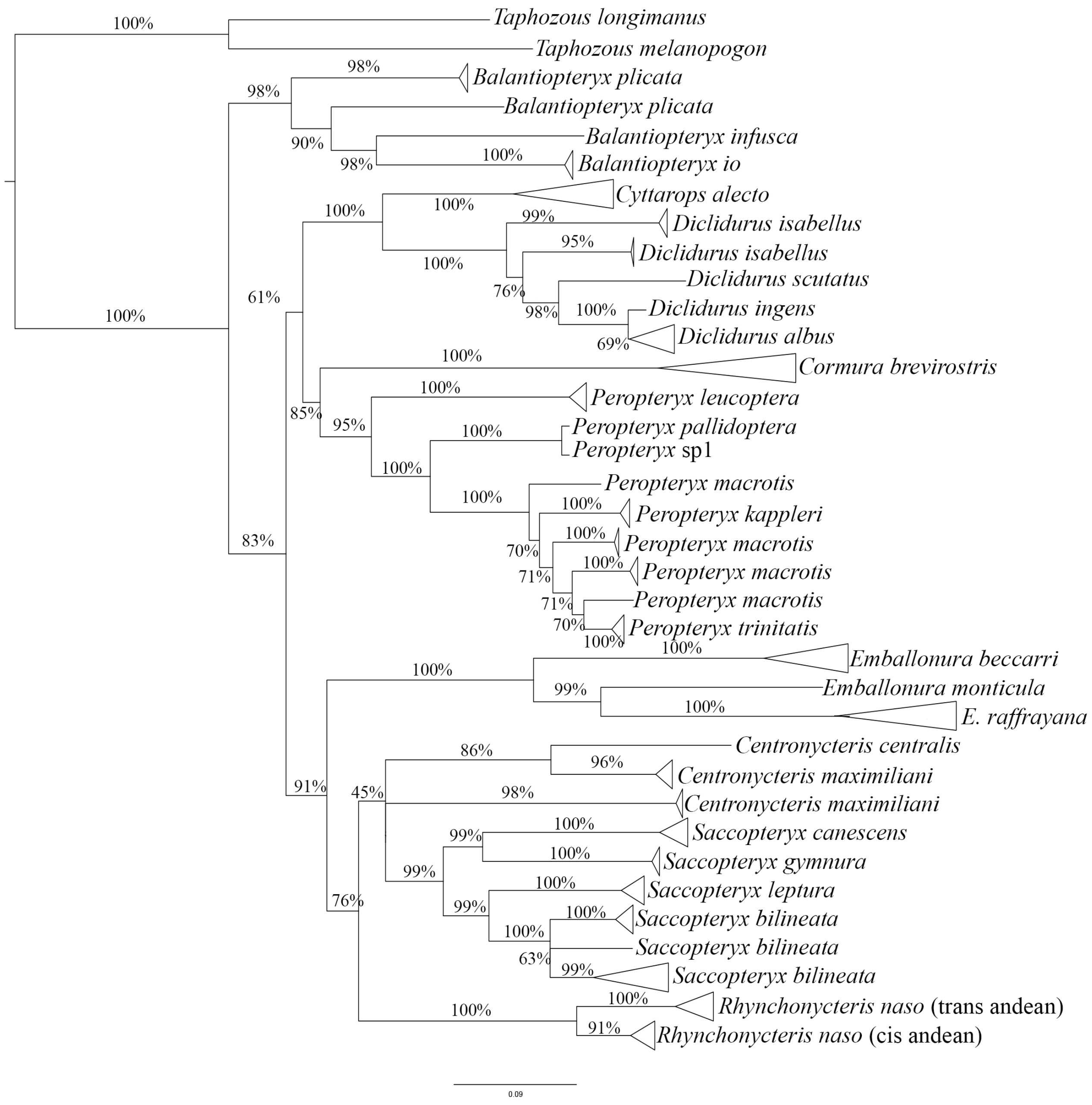

Supplement: S3 Fig — Bootstrap probabilities are indicated at each node. (PDF) [file pone.0285271.s003.pdf]
