# Supplementary material for: Systematics and phylogeography of bats of the genus Rhynchonycteris (Chiroptera: Emballonuridae): Integrating molecular phylogenetics, ecological niche modeling and morphometric data
Source: PLoS One. 2023 May 4;18(5):e0285271. doi: 10.1371/journal.pone.0285271 (PMC10159116; doi:10.1371/journal.pone.0285271)
Supplement: S1 Table — Species with unique identifier number, locality, associated voucher specimen, and GenBank accession number. (PDF) [file pone.0285271.s004.pdf]

Species	Voucher	*Usp9x*	*Chd1*	*Dby*	*Cytb*	*COI*	Country
Balantiopteryx_infusca_1	SP 10792	NA	EF584231	NA	EF584151	NA	Ecuador
Balantiopteryx_infusca_2	SP 10793	NA	NA	EF584297	NA	NA	Ecuador
Balantiopteryx_infusca_3	SP 10794	EF584115	EF584232	NA	NA	NA	Ecuador
Balantiopteryx_io_1	ROM 98425	NA	NA	NA	NA	JF447968	Guatemala
Balantiopteryx_io_2	FN 31415	NA	EF584233	EF584298	EF584152	JF446714	Guatemala
Balantiopteryx_io_3	ROM 99231	EF584114	NA	NA	EF584153	NA	Guatemala
Balantiopteryx_io_4	ROM 98428	NA	NA	NA	NA	JF446715	Guatemala
Balantiopteryx_io_5	ROM 98431	NA	NA	NA	NA	JF446708	Guatemala
Balantiopteryx_io_6	ROM 98434	NA	NA	NA	NA	JF446712	Guatemala
Balantiopteryx_plicata_1	ROM 99654	EF584116	NA	EF584299	EF584154	NA	Guatemala
Balantiopteryx_plicata_2	ROM 98241	NA	EF584234	NA	NA	JF446721	Guatemala
Balantiopteryx_plicata_3	ROM 98237	EF584117	EF584235	NA	NA	JF446719	Guatemala
Balantiopteryx_plicata_4	ROM 99655	NA	EF584236	NA	NA	JF446723	Guatemala
Balantiopteryx_plicata_5	ROM:112202	NA	NA	NA	NA	JF459378	Nicaragua
Balantiopteryx_plicata_6	ROM 98236	NA	NA	NA	NA	JF446722	Guatemala
Centronycteris_centralis_1	CM 119034	EF584129	EF584258	EF584314	EF584155	NA	Guatemala
Centronycteris_maximiliani_1	ROM 115530	EF584131	EF584260	NA	EF584157	NA	Guyana
Centronycteris_maximiliani_2	KU 156014	EF584130	EF584259	NA	EF584156	NA	Guyana
Centronycteris_maximiliani_3	ROM F39343	NA	NA	NA	NA	JF454541	Guyana
Centronycteris_maximiliani_4	ROM 117646	NA	NA	NA	NA	EU096694	Suriname
Centronycteris_maximiliani_5	ROM 117296	NA	NA	NA	NA	EU096693	Suriname
Centronycteris_maximiliani_6	ROM 117543	NA	NA	NA	NA	EU096692	Suriname
Centronycteris_maximiliani_7	ROM 117238	NA	NA	NA	NA	EU096691	Suriname
Cormura_brevirostris_1	ROM 103323	NA	NA	EF584302	NA	NA	Guyana
Cormura_brevirostris_2	ROM 108284	NA	NA	NA	EF584159	JF446592	Costa Rica
Cormura_brevirostris_3	F44026	EF584123	EF584237	EF584300	NA	NA	Costa Rica
Cormura_brevirostris_4	ROM 105746	NA	NA	EF584301	EF584158	JF448838	Ecuador
Cormura_brevirostris_5	CRB2511	NA	NA	MF632302	MF622049	MF622048	Brasil
Cyttarops_alecto_1	ROM 115631	NA	EF584239	NA	EF584161	NA	Guyana
Cyttarops_alecto_2	ROM 113761	NA	NA	EF584303	EF584162	NA	Guyana
Cyttarops_alecto_3	ROM 112626	NA	NA	EF584304	NA	EF080320	Guyana
Cyttarops_alecto_4	ROM 108283	EF584118	NA	NA	EF584160	JF446594	Costa Rica
Cyttarops_alecto_5	ROM 117632	NA	NA	NA	NA	EU096710	Suriname
Diclidurus_albus_1	ROM 107435	EF584119	EF584240	NA	EF584163	JN311813	Guyana
Diclidurus_albus_2	ROM 112292	NA	NA	NA	NA	JN311812	Belize
Diclidurus_ingens_1	ROM 107434	EF584120	EF584241	NA	EF584164	NA	Guyana
Diclidurus_isabellus_1	ROM 107436	EF584122	NA	NA	EF584166	NA	Guyana
Diclidurus_isabellus_2	ROM 108940	NA	EF584243	NA	EF584165	NA	Guyana
Diclidurus_isabellus_3	ROM 109068	NA	NA	EF584305	NA	EF080335	Guyana
Diclidurus_isabellus_4	ROM F39501	NA	NA	NA	NA	JF454655	Guyana
Diclidurus_isabellus_5	ROM F39478	NA	NA	NA	NA	JF454651	Guyana
Diclidurus_isabellus_6	ROM 109103	NA	NA	NA	NA	JF454640	Guyana
Diclidurus_scutatus_1	AMNH 267832	EF584121	EF584242	NA	EF584167	NA	French Guiana
Peropteryx_kappleri_1	AMNH 267834	NA	EF584245	NA	EF584169	NA	French Guiana
Peropteryx_kappleri_2	ROM 100910	NA	EF584244	NA	EF584168	EF080536	Guyana
Peropteryx_kappleri_3	ROM 101123	NA	NA	EF584306	NA	EF080537	Guyana
Peropteryx_kappleri_4	V 1653	NA	NA	NA	HQ693719	NA	
Peropteryx_leucoptera_1	AMNH 267839	NA	EF584253	NA	EF584173	NA	French Guiana

Peropteryx_leucoptera_2	ROM 112531	NA	EF584255	NA	EF584175	EF080539	Guyana
Peropteryx_leucoptera_3	ROM 113612	NA	EF584254	EF584313	EF584174	NA	Guyana
Peropteryx_leucoptera_4	ROM 107458	NA	EF584252	EF584312	EF584172	NA	Guyana
Peropteryx_leucoptera_5	QCAZ 8478	NA	NA	NA	HM367876	NA	Ecuador
Peropteryx_leucoptera_6	ROM 112530	NA	NA	NA	NA	EF080538	Guyana
Peropteryx_leucoptera_7	ROM 113611	NA	NA	NA	NA	EF080540	Guyana
Peropteryx_macrotis_1	ROM 108467	NA	EF584250	EF584309	EF584180	JN311814	Guyana
Peropteryx_macrotis_2	ROM 108523	EF584124	EF584249	NA	EF584179	NA	Guyana
Peropteryx_macrotis_3	ROM 107126	NA	NA	EF584308	EF584178	NA	Guyana
Peropteryx_macrotis_4	FN33843	NA	EF584248	EF584307	EF584177	JF447308	Mexico
Peropteryx_macrotis_5	ROM 96446	EF584125	EF584247	NA	EF584176	JF448098	Mexico
Peropteryx_macrotis_6	ROM 95945	NA	EF584246	NA	NA	JF447307	Mexico
Peropteryx_macrotis_7	ROM 96444	NA	NA	NA	NA	JF447312	Mexico
Peropteryx_macrotis_8	ROM 96443	NA	NA	NA	NA	JF447311	Mexico
Peropteryx_macrotis_9	ROM 96442	NA	NA	NA	NA	JF447310	Mexico
Peropteryx_macrotis_10	ROM 96445	NA	NA	NA	NA	JF447309	Mexico
Peropteryx_macrotis_11	ROM 95946	NA	NA	NA	NA	JF447306	Mexico
Peropteryx_macrotis_12	ROM FN29738	NA	NA	NA	NA	JF447305	Mexico
Peropteryx_macrotis_13	ROM 117527	NA	NA	NA	NA	EU096826	Suriname
Peropteryx_pallidoptera_1	ROM 104396	EF584127	EF584256	EF584311	EF584170	JQ601648	Ecuador
Peropteryx_pallidoptera_2	RSV 2330	EF584128	EF584257	NA	EF584171	NA	French Guiana
Peropteryx_trinitatis_1	ROM 107822	NA	EF584251	NA	EF584181	JF447843	Venezuela
Peropteryx_trinitatis_2	ROM 107920	EF584126	NA	NA	EF584182	JF447840	Venezuela
Peropteryx_trinitatis_3	ROM 107823	NA	NA	EF584310	NA	JF447842	Venezuela
Peropteryx_trinitatis_4	ROM 107916	NA	NA	NA	NA	JF447846	Venezuela
Peropteryx_trinitatis_5	ROM 107830	NA	NA	NA	NA	JF447845	Venezuela
Peropteryx_trinitatis_6	ROM 107921	NA	NA	NA	NA	JF447844	Venezuela
Peropteryx_trinitatis_7	ROM 107825	NA	NA	NA	NA	JF447841	Venezuela
Peropteryx_trinitatis_8	ROM 107831	NA	NA	NA	NA	JF447839	Venezuela
Peropteryx_trinitatis_9	ROM 107922	NA	NA	NA	NA	JF447838	Venezuela
Peropteryx_trinitatis_10	ROM 107923	NA	NA	NA	NA	JF447837	Venezuela
Peropteryx_trinitatis_11	ROM 107824	NA	NA	NA	NA	JF447836	Venezuela
Peropteryx_trinitatis_12	ROM 107918	NA	NA	NA	NA	JF447835	Venezuela
Rhynchonycteris_naso_1	ROM 98769	EF584135	EF584261	NA	EF584190	JF455738	Guyana
Rhynchonycteris_naso_2	ROM 107891	EF584134	EF584263	NA	EF584192	JF447855	Venezuela
Rhynchonycteris_naso_3	ROM 105537	EF584133	EF584262	NA	EF584191	JF449154	Ecuador
Rhynchonycteris_naso_4	ROM 98770	NA	NA	NA	EF584184	JF455739	Guyana
Rhynchonycteris_naso_5	ROM 108102	NA	NA	NA	EF584186	JF455710	Guyana
Rhynchonycteris_naso_6	ROM 112581	EF584132	NA	EF584315	EF584187	JF455704	Guyana
Rhynchonycteris_naso_7	ROM 100444	NA	NA	NA	EF584188.1	JF455736	Guyana
Rhynchonycteris_naso_8	ROM 107264	NA	EF584265	NA	EF584185	JF455718	Guyana
Rhynchonycteris_naso_9	ROM 106583	NA	NA	NA	EF584189	JF455725	Guyana
Rhynchonycteris_naso_10	ROM 97939	NA	EF584264	NA	EF584183	JF455741	Guyana
Rhynchonycteris_naso_11	CM 91643	NA	EF584266	NA	NA	NA	Belize
Rhynchonycteris_naso_14	20120716_33	NA	NA	NA	NA	MG191904	Panama
Rhynchonycteris_naso_15	20120716_31	NA	NA	NA	NA	MG191888	Panama
Rhynchonycteris_naso_16	20120716_26	NA	NA	NA	NA	MG191880	Panama
Rhynchonycteris_naso_17	20120815_217	NA	NA	NA	NA	MG191856	Panama
Rhynchonycteris_naso_18	20120816_175	NA	NA	NA	NA	MG191846	Panama

Rhynchonycteris_naso_19	20120716_27	NA	NA	NA	NA	MG191845	Panama
Rhynchonycteris_naso_20	BCBF-203	NA	NA	NA	NA	JF499035	Belize
Rhynchonycteris_naso_21	BCBF-202	NA	NA	NA	NA	JF499034	Belize
Rhynchonycteris_naso_22	BCBF-201	NA	NA	NA	NA	JF499033	Belize
Rhynchonycteris_naso_23	BCBF-200	NA	NA	NA	NA	JF499032	Belize
Rhynchonycteris_naso_24	ROM 118841	NA	NA	NA	NA	JF449155	Ecuador
Rhynchonycteris_naso_25	ROM 117166	NA	NA	NA	NA	EU096962	Suriname
Rhynchonycteris_naso_26	ROM 117594	NA	NA	NA	NA	EU096960	Suriname
Rhynchonycteris_naso_27	ROM 117600	NA	NA	NA	NA	EU096956	Suriname
Rhynchonycteris_naso_28	ROM 117685	NA	NA	NA	NA	EU096955	Suriname
Rhynchonycteris_naso_29	ROM 116888	NA	NA	NA	NA	JF447725	Suriname
Rhynchonycteris_naso_30	ROM_108952	NA	NA	NA	NA	EF080672	Guyana
Rhynchonycteris_naso_31	ROM_108965	NA	NA	NA	NA	EF080673	Guyana
Rhynchonycteris_naso_32	ROM_108976	NA	NA	NA	NA	EF080674	Guyana
Rhynchonycteris_naso_33	ROM_108977	NA	NA	NA	NA	EF080675	Guyana
Rhynchonycteris_naso_34	ROM_108980	NA	NA	NA	NA	EF080676	Guyana
Rhynchonycteris_naso_35	ROM_117678	NA	NA	NA	NA	EU096954	Suriname
Rhynchonycteris_naso_36	ROM_117164	NA	NA	NA	NA	EU096957	Suriname
Rhynchonycteris_naso_37	ROM_117163	NA	NA	NA	NA	EU096958	Suriname
Rhynchonycteris_naso_38	ROM_117584	NA	NA	NA	NA	EU096959	Suriname
Rhynchonycteris_naso_39	ROM_117165	NA	NA	NA	NA	EU096961	Suriname
Rhynchonycteris_naso_40	ROM_117095	NA	NA	NA	NA	JF447723	Suriname
Rhynchonycteris_naso_41	ROM_117106	NA	NA	NA	NA	JF447724	Suriname
Rhynchonycteris_naso_42	ROM_116887	NA	NA	NA	NA	JF447726	Suriname
Rhynchonycteris_naso_43	ROM_116919	NA	NA	NA	NA	JF447727	Suriname
Rhynchonycteris_naso_44	ROM_116918	NA	NA	NA	NA	JF447728	Suriname
Rhynchonycteris_naso_45	ROM_111568	NA	NA	NA	NA	JF455672	Guyana
Rhynchonycteris_naso_46	ROM_112645	NA	NA	NA	NA	JF455673	Guyana
Rhynchonycteris_naso_47	ROM_111567	NA	NA	NA	NA	JF455674	Guyana
Rhynchonycteris_naso_48	ROM_112624	NA	NA	NA	NA	JF455675	Guyana
Rhynchonycteris_naso_49	ROM_112622	NA	NA	NA	NA	JF455676	Guyana
Rhynchonycteris_naso_50	ROM_108966	NA	NA	NA	NA	JF455677	Guyana
Rhynchonycteris_naso_51	ROM_109152	NA	NA	NA	NA	JF455678	Guyana
Rhynchonycteris_naso_52	ROM_109220	NA	NA	NA	NA	JF455679	Guyana

Rhynchonycteris_naso_53	ROM_109146	NA	NA	NA	NA	JF455680	Guyana
Rhynchonycteris_naso_54	ROM_109183	NA	NA	NA	NA	JF455681	Guyana
Rhynchonycteris_naso_55	ROM_109066	NA	NA	NA	NA	JF455682	Guyana
Rhynchonycteris_naso_56	ROM_109211	NA	NA	NA	NA	JF455683	Guyana
Rhynchonycteris_naso_57	ROM_109206	NA	NA	NA	NA	JF455684	Guyana
Rhynchonycteris_naso_58	ROM_109205	NA	NA	NA	NA	JF455685	Guyana
Rhynchonycteris_naso_59	ROM_108979	NA	NA	NA	NA	JF455686	Guyana
Rhynchonycteris_naso_60	ROM_108978	NA	NA	NA	NA	JF455687	Guyana
Rhynchonycteris_naso_61	ROM_119039	NA	NA	NA	NA	JF455688	Guyana
Rhynchonycteris_naso_62	ROM_119038	NA	NA	NA	NA	JF455689	Guyana
Rhynchonycteris_naso_63	ROM_115672	NA	NA	NA	NA	JF455690	Guyana
Rhynchonycteris_naso_64	ROM_119129	NA	NA	NA	NA	JF455691	Guyana
Rhynchonycteris_naso_65	ROM_119093	NA	NA	NA	NA	JF455692	Guyana
Rhynchonycteris_naso_66	ROM_115733	NA	NA	NA	NA	JF455693	Guyana
Rhynchonycteris_naso_67	ROM_115732	NA	NA	NA	NA	JF455694	Guyana
Rhynchonycteris_naso_68	ROM_119075	NA	NA	NA	NA	JF455695	Guyana
Rhynchonycteris_naso_69	ROM_113779	NA	NA	NA	NA	JF455696	Guyana
Rhynchonycteris_naso_70	ROM_113778	NA	NA	NA	NA	JF455697	Guyana
Rhynchonycteris_naso_71	ROM_111709	NA	NA	NA	NA	JF455698	Guyana
Rhynchonycteris_naso_72	ROM_112582	NA	NA	NA	NA	JF455699	Guyana
Rhynchonycteris_naso_73	ROM_112618	NA	NA	NA	NA	JF455700	Guyana
Rhynchonycteris_naso_74	ROM_111610	NA	NA	NA	NA	JF455701	Guyana
Rhynchonycteris_naso_75	ROM_111733	NA	NA	NA	NA	JF455702	Guyana
Rhynchonycteris_naso_76	ROM_112623	NA	NA	NA	NA	JF455703	Guyana
Rhynchonycteris_naso_77	ROM_111595	NA	NA	NA	NA	JF455705	Guyana
Rhynchonycteris_naso_78	ROM_106729	NA	NA	NA	NA	JF455706	Guyana
Rhynchonycteris_naso_79	ROM_107370	NA	NA	NA	NA	JF455707	Guyana
Rhynchonycteris_naso_80	ROM_106730	NA	NA	NA	NA	JF455708	Guyana
Rhynchonycteris_naso_81	ROM_F39757	NA	NA	NA	NA	JF455709	Guyana

Rhynchonycteris_naso_82	ROM_106637	NA	NA	NA	NA	JF455711	Guyana
Rhynchonycteris_naso_83	ROM_106731	NA	NA	NA	NA	JF455712	Guyana
Rhynchonycteris_naso_84	ROM_108103	NA	NA	NA	NA	JF455713	Guyana
Rhynchonycteris_naso_85	ROM_106638	NA	NA	NA	NA	JF455714	Guyana
Rhynchonycteris_naso_86	ROM_108104	NA	NA	NA	NA	JF455715	Guyana
Rhynchonycteris_naso_87	ROM_106552	NA	NA	NA	NA	JF455716	Guyana
Rhynchonycteris_naso_88	ROM_106607	NA	NA	NA	NA	JF455717	Guyana
Rhynchonycteris_naso_89	ROM_107265	NA	NA	NA	NA	JF455719	Guyana
Rhynchonycteris_naso_90	ROM_107125	NA	NA	NA	NA	JF455720	Guyana
Rhynchonycteris_naso_91	ROM_106712	NA	NA	NA	NA	JF455721	Guyana
Rhynchonycteris_naso_92	ROM_107302	NA	NA	NA	NA	JF455722	Guyana
Rhynchonycteris_naso_93	ROM_106582	NA	NA	NA	NA	JF455723	Guyana
Rhynchonycteris_naso_94	ROM_107404	NA	NA	NA	NA	JF455724	Guyana
Rhynchonycteris_naso_95	ROM_106584	NA	NA	NA	NA	JF455726	Guyana
Rhynchonycteris_naso_96	ROM_106688	NA	NA	NA	NA	JF455727	Guyana
Rhynchonycteris_naso_97	ROM_108099	NA	NA	NA	NA	JF455728	Guyana
Rhynchonycteris_naso_98	ROM_106726	NA	NA	NA	NA	JF455729	Guyana
Rhynchonycteris_naso_99	ROM_108100	NA	NA	NA	NA	JF455730	Guyana
Rhynchonycteris_naso_100	ROM_98093	NA	NA	NA	NA	JF455731	Guyana
Rhynchonycteris_naso_101	ROM_98796	NA	NA	NA	NA	JF455732	Guyana
Rhynchonycteris_naso_102	ROM_98094	NA	NA	NA	NA	JF455733	Guyana
Rhynchonycteris_naso_103	ROM_98095	NA	NA	NA	NA	JF455734	Guyana
Rhynchonycteris_naso_104	ROM_98016	NA	NA	NA	NA	JF455735	Guyana
Rhynchonycteris_naso_105	ROM_100445	NA	NA	NA	NA	JF455737	Guyana
Rhynchonycteris_naso_106	ROM_97821	NA	NA	NA	NA	JF455740	Guyana
Rhynchonycteris_naso_107	ROM_119819	NA	NA	NA	NA	JF459272	Guyana
Rhynchonycteris_naso_108	ROM_119801	NA	NA	NA	NA	JF459273	Guyana
Rhynchonycteris_naso_109	ROM_116965	NA	NA	NA	NA	JQ601313	Suriname
Saccopteryx_bilineata_1	ROM 105522	EF584137	NA	EF584316	NA	JF448131	Ecuador
Saccopteryx_bilineata_2	ROM 105672	NA	EF584270	NA	EF584197	JF435737	Ecuador
Saccopteryx_bilineata_3	ROM 101336	NA	NA	NA	NA	JF448129	El Salvador
Saccopteryx_bilineata_4	ROM 101337	NA	EF584269	NA	EF584193	JF435740	El Salvador
Saccopteryx_bilineata_5	ROM 99323	EF584138	EF584268	NA	EF584201	JF435741	Guatemala
Saccopteryx_bilineata_6	ROM 113740	NA	NA	EF584317	NA	NA	Guyana
Saccopteryx_bilineata_7	ROM 115534	NA	EF584274	NA	EF584202	NA	Guyana

Saccopteryx_bilineata_8	ROM 106613	NA	EF584273	NA	NA	JF435712	Guyana
Saccopteryx_bilineata_9	ROM 97921	NA	EF584272	JF458506	EF584199	JF435725	Guyana
Saccopteryx_bilineata_10	ROM 96376	NA	EF584267	NA	EF584198	JF448126	Mexico
Saccopteryx_bilineata_11	ROM 113893	NA	NA	NA	EF584194	JF435733	Suriname
Saccopteryx_bilineata_12	ROM 114081	NA	EF584275	NA	EF584195	NA	Suriname
Saccopteryx_bilineata_13	ROM 114083	EF584136	EF584276	EF584318	EF584196	NA	Suriname
Saccopteryx_bilineata_14	ROM 107914	NA	EF584271	EF584319	EF584200	JF448130	Venezuela
Saccopteryx_bilineata_15	ROM F37639	NA	NA	JF458504	NA	JF435718	Ecuador
Saccopteryx_bilineata_16	ROM 105363	NA	NA	JF458505	NA	JF435720	Ecuador
Saccopteryx_bilineata_17	ROM 103255	NA	NA	JF458472	NA	JF435659	Guyana
Saccopteryx_bilineata_18	ROM 103256	NA	NA	JF458473	NA	JF435660	Guyana
Saccopteryx_bilineata_19	ROM 103303	NA	NA	JF458474	NA	JF435661	Guyana
Saccopteryx_bilineata_20	ROM 114141	NA	NA	JF458507	NA	JF435735	Suriname
Saccopteryx_bilineata_21	20120411_331	NA	NA	NA	NA	MG191881	Panama
Saccopteryx_bilineata_22	20130817_519	NA	NA	NA	NA	MG191794	Panama
Saccopteryx_bilineata_23	ROM 114138	NA	NA	NA	NA	JF435736	Suriname
Saccopteryx_bilineata_24	ROM 113980	NA	NA	NA	NA	JF435734	Suriname
Saccopteryx_bilineata_25	ROM 99322	NA	NA	NA	NA	JF435731	Guatemala
Saccopteryx_bilineata_26	AVB080324-02	NA	NA	NA	NA	HM208681	Mexico
Saccopteryx_canescens_1	ROM 100446	NA	EF584285	NA	EF584205	EF080679	Guyana
Saccopteryx_canescens_2	ROM 100207	EF584144	NA	NA	EF584204	NA	Guyana
Saccopteryx_canescens_3	ROM 97899	EF584143	EF584286	NA	EF584203	NA	Guyana
Saccopteryx_canescens_4	ROM 107890	NA	EF584287	EF584325	EF584206	JF447856	Venezuela
Saccopteryx_gymnura_1	AMNH 267843	NA	EF584289	NA	EF584208	NA	Fench Guiana
Saccopteryx_gymnura_2	ROM 102952	EF584145	EF584288	NA	EF584207	EF080680	Guyana
Saccopteryx_gymnura_3	ROM 117563	NA	NA	NA	NA	EU097001	Suriname
Saccopteryx_gymnura_4	ROM 117094	NA	NA	NA	NA	JQ601314	Suriname
Saccopteryx_leptura_1	ROM 105530	EF584140	EF584278	NA	NA	JF449156	Ecuador
Saccopteryx_leptura_2	ROM 105531	NA	NA	EF584321	EF584213	JF449157	Ecuador
Saccopteryx_leptura_3	ROM 113741	NA	EF584283	NA	NA	NA	Guyana
Saccopteryx_leptura_4	ROM 100227	NA	NA	EF584322	NA	EF080681	Guyana
Saccopteryx_leptura_5	ROM 108941	NA	EF584281	NA	EF584214	NA	Guyana
Saccopteryx_leptura_6	ROM 103356	NA	EF584280	NA	EF584211	NA	Guyana
Saccopteryx_leptura_7	ROM 97920	NA	EF584279	NA	EF584210	JF455752	Guyana
Saccopteryx_leptura_8	ROM 104208	EF584139	EF584277	EF584320	EF584212	JF447433	Panama
Saccopteryx_leptura_9	ROM 113878	NA	EF584282	EF584324	EF584216	NA	Suriname
Saccopteryx_leptura_10	ROM 114190	EF584142	EF584284	NA	EF584217	NA	Suriname
Saccopteryx_leptura_11	ROM 114223	NA	NA	NA	EF584209	JF447729	Suriname
Saccopteryx_leptura_12	ACUNHC 366	EF584141	NA	EF584323	EF584215	NA	Venezuela
Saccopteryx_leptura_13	ROM 115598	NA	NA	NA	NA	JF455749	Guyana
Saccopteryx_leptura_14	ROM 106574	NA	NA	NA	NA	JF455751	Guyana
Saccopteryx_leptura_15	ROM 112047	NA	NA	NA	NA	JF455750	Guyana
Saccopteryx_leptura_16	ROM MAM 120022	NA	NA	NA	NA	HQ545503	Suriname
Saccopteryx_leptura_17	ROM 116938	NA	NA	NA	NA	JQ601312	Suriname
Saccopteryx_leptura_18	ROM 114240	NA	NA	NA	NA	JF447730	Suriname
Emballonura_beccarrii_1	TK 20327	NA	EF584290	EF584326	EF584222	NA	Papua New Guinea
Emballonura_beccarrii_2	Australian	NA	NA	NA	EF635537	EF635573	Papua New

	Museum M17969						Guinea
Emballonura_beccarrii_3	Australian Museum M19406	NA	NA	NA	NA	EF635570	Papua New Guinea
Emballonura_monticola_1	TK 21373	NA	EF584291	NA	EF584223	NA	Thailand
Emballonura_raffrayana_1	Australian Museum M19847	NA	NA	NA	EF635545	EF635580	Papua New Guinea
Emballonura_raffrayana_2	Australian Museum M23380	NA	NA	NA	EF635560	EF635588	Papua New Guinea
Mossia_nigrescens_1	D82	EF584146	NA	EF584327	NA	NA	Papua New Guinea
Taphozous_longimanus_1	ROM 107743	EF584148	NA	NA	EF584218	HM541967	Vietnam
Taphozous_melanopogon_1	ROM 110979	NA	EF584292	NA	EF584221	NA	Vietnam

Morphometric analysis: specimens examined

ID	Sex	Country	Longitude	Latitude	Andean position
UFES-4323	UNK	BR	-40.147430556	-19.055722222	CIS
MZUSP-22671	F	BR	-52.533449	-3.8013	CIS
MZUSP-22732	F	BR	-52.533449	-3.8013	CIS
MZUSP-22703	F	BR	-52.533449	-3.8013	CIS
MZUSP-22693	M	BR	-52.533449	-3.8013	CIS
MZUSP-2670	F	BR	-43.145381	-11.096473	CIS
MZUSP-2669	M	BR	-43.145381	-11.096473	CIS
MZUSP-5614	M	BR	-44.854594	-4.662916	CIS
MZUSP-5625	F	BR	-44.854594	-4.662916	CIS
MZUSP-2430	F	BR	-39.822482	-19.644763	CIS
MZUSP-2429	M	BR	-39.822482	-19.644763	CIS
MZUSP-7398	UNK	BR	-38.501648	-12.977733	CIS
MZUSP-30118	F	BR	-44.166667	-8.833333	CIS
UIS-MHN-M-780	M	CO	-73.383639	6.999611	TRANS
UIS-MHN-M-1074	M	CO	-73.357919	7.26743	TRANS
UIS-MHN-M-1386	M	CO	-73.357917	7.267417	TRANS
UIS-MHN-M-1749	F	CO	-74.20261	7.053805	TRANS
UIS-MHN-M-1768	F	CO	-74.1969	7.06225	TRANS
IAVH-M-2481	M	CO	-67.84	5.345	CIS
IAVH-M-2480	F	CO	-73.794158	2.264193	CIS
IAVH-M-2025	F	CO	-73.925	2.519	CIS
IAVH-M-0695	F	CO			CIS
IAVH-M-4906	F	CO			TRANS
IAVH-M-4907	M	CO			TRANS
IAVH-M-0959	M	CO			TRANS
IAVH-M-2030	M	CO	-73.925	2.519	CIS
M-248745	M	BO			CIS
M-209189	M	BO			CIS
M-210458	M	BO			CIS
M-210457	F	BO			CIS
MO-9560	UNK	CR			TRANS

MO-9559	UNK	CR			TRANS
M-71506	M	EC			CIS
M-71505	M	EC			CIS
M-67605	F	EC			CIS
M-67604	F	EC			CIS
M-265988	F	GF			CIS
M-267373	F	GF			CIS
M-267372	M	GF			CIS
M-182728	M	GY			CIS
M-34888	UNK	GY			CIS
M-34893	UNK	GY			CIS
M-142830	UNK	GY			CIS
MS-7439	F	TT			CIS
MS-7438	UNK	TT			CIS
M-176618	M	TT			CIS
ICN-17205	M	CO	-75.8344	9.2333	TRANS
ICN-17206	F	CO	-75.8344	9.2333	TRANS
ICN-17210	F	CO			TRANS
ICN-17211	M	CO			TRANS
ICN-873	M	CO			TRANS
ICN-14973-83	F	CO	-67.8522	3.6544	CIS
ICN-22640	F	CO	-72.6956	2.5711	CIS
ICN-22641	M	CO	-72.7022	2.5711	CIS
ICN-22546	F	CO	-72.7022	2.5711	CIS
ICN-8503	M	CO			CIS
ICN-14802	M	CO			TRANS
ICN-1398	F	CO			CIS
ICN-2024	M	CO			CIS
ICN-11354	M	CO	-73.451044	4.259676	CIS
ICN-11357	M	CO	-73.451044	4.259676	CIS
ICN-18757	M	CO	-73.9693	6.6125	TRANS
ICN-18758	F	CO	-73.9097	6.6125	TRANS
ICN-18759	F	CO	-73.9693	6.6125	TRANS
ICN-18760	F	CO	-73.9693	6.6125	TRANS
ICN-21121	M	CO			TRANS
ICN-5870	M	CO			TRANS
ICN-13110	F	CO			CIS
ICN-13111	F	CO			CIS
ICN-17922	F	CO	-69.5381	-1.0878	CIS
ICN-17923	F	CO	-69.5056	-1.1083	CIS
ICN-19264	M	CO	-71.4247	5.4747	CIS
ICN-24583	F	CO	-71.411317	5.273844	CIS
ICN-22932	M	CO			CIS
ICN-11291	M	CO			CIS
ICN-14613	M	CO			CIS
ICN-21470	M	CO	-75.36748	1.49031	CIS
ICN-11282	M	CO			CIS
ICN-11283	F	CO			CIS
ICN-11285	M	CO			CIS
ICN-11286	M	CO			CIS
ICN-11287	M	CO			CIS
ICN-23170	M	CO	-73.8	2.2666	CIS
ICN-23169	M	CO	-73.75	2.4667	CIS

ICN-11358	F	CO	-73.451044	4.259676	CIS
ICN-13901	F	CO	-73.55605	4.26866	CIS
ICN-11359	F	CO	-73.451044	4.259676	CIS
ICN-11360	F	CO	-73.451044	4.259676	CIS
ICN-13902	F	CO	-73.55605	4.26866	CIS
ICN-18385	M	CO	-71.3333	4.5667	CIS
QCAZ-6959	M	EC	-75.7662	-0.31812	CIS
QCAZ-001	F	EC	-79.3867	0.00417	TRANS
QCAZ236	M	EC	-79.3867	0.00417	TRANS
MUSM-27163	F	PE	-73.27172	-3.70095	CIS
MUSM-25-7-153	M	PE			CIS
MUSM-1082	F	PE	-70.583	-13.25	CIS
MUSM-14800	F	PE	-72.86667	-11.8	CIS
MUSM-6745	M	PE	-72.91738	-3.25721	CIS
MUSM-24579	M	PE	-74.93567	-9.61369	CIS
MUSM-1095	M	PE	-74.6	-8.3	CIS
MUSM-20975	F	PE	-75.2596	-0.52827	CIS
MUSM-20976	M	PE	-75.2596	-0.52827	CIS
MUSM-20977	M	PE	-75.2596	-0.52827	CIS
MUSM-1093	M	PE	-74.6	-8.3	CIS
MUSM-1089	F	PE	-74.6	-8.3	CIS
MUSM-1090	F	PE	-74.53042	-8.37844	CIS
MUSM-1092	M	PE	-74.6	-8.3	CIS
MUSM-1091	F	PE	-74.6	-8.3	CIS
MUSM-1094	M	PE	-74.6	-8.3	CIS
MUSM-10331	M	PE	-75.26194	-10.32222	CIS
MUSM-493	M	PE	-75.25	-10.16667	CIS
MUSM-494	F	PE	-75.25	-10.16667	CIS
MUSM-1085	M	PE	-71.21667	-10.13333	CIS
MUSM-27151	M	PE	-73.3567	-3.784	CIS
MUSM-12581	M	PE	-71.2833	-11.9464	CIS
MUSM-11707	F	PE	-68.88167	-12.95667	CIS
MUSM-6783	M	PE	-71.2833	-11.9464	CIS
MUSM-11706	M	PE	-68.88167	-12.95667	CIS
MUSM-24174	M	PE	-75.24	-10.38	CIS
MUSM-12852	M	PE	-68.653	-12.502	CIS
MUSM-1087	M	PE	-69.296	-12.837	CIS

Morphometric analysis: specimens examined (Continuation)

ID	Country	State	County	Locality
UFES-4323	BR	Espírito Santo	Sooretama	Reserva Biológica de Sooretama
MZUSP-22671	BR	Para		Cachoeira do espelho, Rio Xingu
MZUSP-22732	BR	Para		Cachoeira do espelho, Rio Xingu
MZUSP-22703	BR	Para		Cachoeira do espelho, Rio Xingu
MZUSP-	BR	Para		Cachoeira do espelho, Rio Xingu

22693				
MZUSP-2670	BR	Bahia		Cidade da Barra
MZUSP-2669	BR	Bahia		Cidade da Barra
MZUSP-5614	BR	Maranhão		Igarape Grande, Rio Jurua
MZUSP-5625	BR	Maranhão		Igarape Grande, Rio Jurua
MZUSP-2430	BR	Espírito Santo		Rio doce
MZUSP-2429	BR	Espírito Santo		Rio doce
MZUSP-7398	BR	Bahia		Salvador
MZUSP-30118	BR	Piaui		Estçao ecologica de uruçui, Una, Bom Jesus
UIS-MHN-M-780	CO	Santander		Vda. Cananá, zapatoca
UIS-MHN-M-1074	CO	Santander		Lebrija, Uribe
UIS-MHN-M-1386	CO	Santander		Lebrija, Uribe
UIS-MHN-M-1749	CO	Bolivar		Cantagallo
UIS-MHN-M-1768	CO	Bolivar		Cantagallo
IAVH-M-2481	CO	Vichada	Cumaribo	PNN El Tuparro, 2 Km SE de Centro Administrativo
IAVH-M-2480	CO	Meta	La Macarena	Arriba caño Cristales
IAVH-M-2025	CO	Meta	La Macarena	PNN La Macarena, Cabaña Duda
IAVH-M-0695	CO	Amazonas	La Chorrera	Cacerio Kuiru, 15 Km abajo de La Chorrera Río Igara-Paraná
IAVH-M-4906	CO	Chocó	Riosucio	PNN Los Katios, Vereda Cristales
IAVH-M-4907	CO	Chocó	Riosucio	PNN Los Katios, Vereda Cristales
IAVH-M-0959	CO	Antioquia	Zaragoza	25 Km al sur y 22 Km al oeste de Zaragoza, La Tirana
IAVH-M-2030	CO	Meta	La Macarena	PNN La Macarena, Cabaña Duda
M-248745	BO	Pando	Nicolas Suarez	Nareuda River
M-209189	BO	Beni	Mamore	Baures River mouth
M-210458	BO	Beni	Cercado	ca. 4 kilometers from Tijamuchi River mouth
M-210457	BO	Beni	General Jose Ballivian	ca. 6 kilometers south of Buena Hora, Arroyo Mercedes
MO-9560	CR	Cartago	Cartago	Irazu Volcano
MO-9559	CR	Cartago	Cartago	Irazu Volcano
M-71506	EC	Napo		Lagartococha River mouth
M-71505	EC	Napo		Lagartococha River mouth
M-67605	EC	Pastaza		Bobonaza River
M-67604	EC	Pastaza		Bobonaza River
M-265988	GF	Cayenne	Sinnamary	Paracou
M-267373	GF	Cayenne	Sinnamary	Paracou
M-267372	GF	Cayenne	Sinnamary	Paracou
M-182728	GY	Upper Takutu - Upper Essequibo	Dadanawa	Rupununi Savanna

M-34888	GY	Region Cuyuni - Mazaruni Region		Potaro River, above Kaieteur Falls
M-34893	GY	Cuyuni - Mazaruni Region		Potaro River, above Kaieteur Falls
M-142830	GY	Cuyuni - Mazaruni Region		Kartabo Point
MS-7439	TT	Trinidad	Caroni County	Caroni River
MS-7438	TT	Trinidad	Caroni County	Caroni River
M-176618	TT	Trinidad	Saint Patrick County	Granville Village
ICN-17205	CO	Cordoba		Mpio Lorica, Est. piscicola de la CVS
ICN-17206	CO	Cordoba		Mpio Lorica, Est. piscicola de la CVS
ICN-17210	CO	Cordoba		Mpio Lorica, Corg. Narino, cienaga pantano bonito
ICN-17211	CO	Cordoba		Mpio Lorica, Vda. Tejada, cienaga pantano bonito
ICN-873	CO	Magdalena		Colonia agricola de caracolito
ICN-14973-83	CO	Guainia		Pto. Inirida, Canho Caiman, resguardo el remanso
ICN-22640	CO	Guaviare		Mpio.San Jose del Guaviare, Playa Guio, Canho negro
ICN-22641	CO	Guaviare		Mpio.San Jose del Guaviare, Playa Guio, Canho negro
ICN-22546	CO	Guaviare		Mpio.San Jose del Guaviare, Playa Guio, Canho negro
ICN-8503	CO	Amazonas		Leticia, Pto. Santander, corregimiento, Pto. Santander, alrededor caserio Araracuara
ICN-14802	CO	Boyaca		Mpio. Puerto Boyaca, Km 30 via Pto. Romero al El Ocal. 5 Km de Techint.
ICN-1398	CO	Meta		Apiay, en bosquesito
ICN-2024	CO	Meta		Canho Yurimena
ICN-11354	CO	Meta		Cumaral, la Y puente sobre via Paratebueno, Medina-Cumaral, +- 3 Km NW
ICN-11357	CO	Meta	Cumaral	Vereda Cepera, 5 Km. NE de Cumaral, La Ye, quebrada Seca.
ICN-18757	CO	Santander	Puerto Parra	Vda. India baja, Corq campo capote, lago Proyecto, Carrare-Opon
ICN-18758	CO	Santander	Puerto Parra	Vda. India baja, Corq campo capote, lago Proyecto, Carrare-Opon
ICN-18759	CO	Santander	Puerto Parra	Vda. India baja, Corq campo capote, lago Proyecto, Carrare-Opon
ICN-18760	CO	Santander	Puerto Parra	Vda. India baja, Corq campo capote, lago Proyecto, Carrare-Opon
ICN-21121	CO	Santander	Sabana de Torres	Finca Villa Natalia
ICN-5870	CO	Valle del Cauca	Buenaventura	Quebrada Zabaleta, aproximadamente 10 Km. Al Norte de Zabaleta
ICN-13110	CO	Vaupés	Mitú	A orillas del rio Cuduiari.
ICN-13111	CO	Vaupés	Mitú	A orillas del rio Cuduiari.
ICN-17922	CO	Vaupés		Est. Caparu, bajo rio Apoporis
ICN-17923	CO	Vaupés		Est. Caparu, bajo rio Apoporis
ICN-19264	CO	Casanare	Trinidad	Vda. Los chochos, Finca las Plumas
ICN-24583	CO	Casanare	San Luis de Palenque	Vda. La nevera, finca Britania
ICN-22932	CO	Casanare	San Luis de Palenque	Vda. Guaracura, Finca los recuerdos
ICN-11291	CO	Caquetá	La Montañita	Cercanias escuela Palma Azul, quebrada Coconuco.

ICN-14613	CO	Caquetá		Rio Mesay, Puerto abeja (rebalse), al suroriende de serrania de chiribiquete
ICN-21470	CO	Caquetá	La Montañita	Vda. Juntas, Hda. Las Delicias, puente concor
ICN-11282	CO	Caquetá	La Montañita	Cercanias escuela Palma Azul, quebrada Coconuco.
ICN-11283	CO	Caquetá	La Montañita	Cercanias escuela Palma Azul, quebrada Coconuco.
ICN-11285	CO	Caquetá	La Montañita	Cercanias escuela Palma Azul, quebrada Coconuco.
ICN-11286	CO	Caquetá	La Montañita	Cercanias escuela Palma Azul, quebrada Coconuco.
ICN-11287	CO	Caquetá	La Montañita	Cercanias escuela Palma Azul, quebrada Coconuco.
ICN-23170	CO	Meta	La Macarena	Vda. Canho cristales, PNN Sierra de la macarena
ICN-23169	CO	Meta	La Macarena	Vda. Canho cristales, PNN Sierra de la macarena
ICN-11358	CO	Meta	Cumaral	Vereda Cepera, 5 Km. NE de Cumaral, La Ye, quebrada Seca.
ICN-13901	CO	Meta	Restrepo	Centro CREAD (Unillanos), en bosque detras de las instalaciones, vereda El Palmar, via a San Nicolas, Km. 20 caño Caibe.
ICN-11359	CO	Meta	Cumaral	Vereda Cepera, 5 Km. NE de Cumaral, La Ye, quebrada Seca.
ICN-11360	CO	Meta	Cumaral	Vereda Cepera, 5 Km. NE de Cumaral, La Ye, quebrada Seca.
ICN-13902	CO	Meta	Restrepo	Centro CREAD (Unillanos), en bosque detras de las instalaciones, vereda El Palmar, via a San Nicolas, Km. 20 caño Caibe.
ICN-18385	CO	Meta		Pto Gaitan, Centro de investigacion Carimagua
QCAZ-6959	EC	Sucumbíos		Zábalo, Terrenos aledaños (al oeste) de la Comunidad de Zábalo
QCAZ-001	EC	Santo Domingo de los Tsáchilas		Bosque Protector La Perla
QCAZ236	EC	Santo Domingo de los Tsáchilas		Bosque Protector La Perla
MUSM-27163	PE	Loreto	Maynas	Punchana, Río Nanay, Puerto Transelva
MUSM-25-7-153	PE	Loreto	Maynas	Corrientillo, Km. 6 de la carretera Iquitos-Nauta, 4.5 Km W camino aZungarococha
MUSM-1082	PE	Cuzco	Quispicanchi	Camanti, Marcapata, Huajyumbe
MUSM-14800	PE	Cuzco	La Convención	Echarate, La Convención, Camisea, Konkariari
MUSM-6745	PE	Loreto	Maynas	Mazán, Qa- Sucusari, Explonapo Camp
MUSM-24579	PE	Huánuco	Puerto Inca	Yuyapichis, Est. Biológica Panguana
MUSM-1095	PE	Ucayali	Coronel Portillo	Yarinacocha, Yarinacocha
MUSM-20975	PE	Loreto	Maynas	Torres Causana, Río Lagartococha, Campamento Catalino
MUSM-20976	PE	Loreto	Maynas	Torres Causana, Río Lagartococha, Campamento Catalino
MUSM-20977	PE	Loreto	Maynas	Torres Causana, Río Lagartococha, Campamento Catalino
MUSM-1093	PE	Ucayali	Coronel Portillo	Yarinacocha, Pucallpa, Yarinacocha
MUSM-1089	PE	Ucayali	Coronel Portillo	Yarinacocha, Yarinacocha, R Ucayali
MUSM-1090	PE	Ucayali	Coronel Portillo	Callaria, Pucallpa
MUSM-1092	PE	Ucayali	Coronel Portillo	Yarinacocha, Yarinacocha, R Ucayali
MUSM-1091	PE	Ucayali	Coronel Portillo	Yarinacocha, Yarinacocha, R Ucayali
MUSM-1094	PE	Ucayali	Coronel Portillo	Yarinacocha, Pucallpa, Yarinacocha
MUSM-10331	PE	Pasco	Oxapampa	Palcazú, Est. Biol. Paujil (P.N. Yanachaga-Chemillen)
MUSM-493	PE	Pasco	Oxapampa	Palcazú, Huancabamba, Comunidad Nativa Castillo

MUSM-494	PE	Pasco	Oxapampa	Palcazú, Huancabamba, Comunidad Nativa Castillo
MUSM-1085	PE	Ucayali	Purús	Purús, R Curanja, Balta
MUSM-27151	PE	Loreto	Maynas	San Juan Bautista, Caserío Santa Sofía, río Nanay (playa)
MUSM-12581	PE	Madre de Dios	Manu	Manu, P.N. Manu, Est. Biol. Pakitza
MUSM-11707	PE	Madre de Dios	Tambopata	Tambopata, S.N. Pampas del Heath, Refugio Juliaca, 12°57,4'S; 68°52,9'W
MUSM-6783	PE	Madre de Dios	Manu	Manu, Manu, Pakitza
MUSM-11706	PE	Madre de Dios	Tambopata	Tambopata, S.N. Pampas del Heath, Refugio Juliaca, 12°57,4'S; 68°52,9'W
MUSM-24174	PE	Pasco	Oxapampa	Palcazú, Río Pescado
MUSM-12852	PE	Madre de Dios	Tambopata	S.N Pampas del Heath
MUSM-1087	PE	Madre de Dios	Tambopata	Tambopata, Explorer's Inn Lodge
