# Supplementary material for: Systematics and phylogeography of bats of the genus Rhynchonycteris (Chiroptera: Emballonuridae): Integrating molecular phylogenetics, ecological niche modeling and morphometric data
Source: PLoS One. 2023 May 4;18(5):e0285271. doi: 10.1371/journal.pone.0285271 (PMC10159116; doi:10.1371/journal.pone.0285271)
Supplement: S2 Table — The numbers following the species names correspond to the unique identifiers. (PDF) [file pone.0285271.s005.pdf]

Specie	Population	Longitude	Latitude	Haplotypes	N° Museum	N° GenBank
*R_naso*_1	S33	-59.23	7.67	Hap1	ROM 98769	JF455738
*R_naso*_4	S33	-59.23	7.67	Hap1	ROM 98770	JF455739
*R_naso*_2	S30	-67.42	6.05	Hap2	ROM 107891	JF447855
*R_naso*_3	S29	-76.35	-0.83	Hap3	ROM 105537	JF449154
*R_naso*_68	S11	-59.53	2.83	Hap3	ROM_119075	JF455695
*R_naso*_93	S10	-58.77	1.37	Hap3	ROM_106582	JF455723
*R_naso*_5	S23	-60.65	6.05	Hap4	ROM 108102	JF455710
*R_naso*_86	S23	-60.65	6.05	Hap4	ROM_108104	JF455715
*R_naso*_6	S9	-58.22	6.5	Hap5	ROM 112581	JF455704
*R_naso*_7	S26	-57.3	5.17	Hap6	ROM 100444	JF455736
*R_naso*_98	S20	-58.83	1.53	Hap6	ROM_106726	JF455729
*R_naso*_8	S17	-58.85	4.73	Hap7	ROM 107264	JF455718
*R_naso*_9	S10	-58.77	1.37	Hap8	ROM 106583	JF455725
*R_naso*_10	S21	-59.3	3.75	Hap9	ROM 97939	JF455741
*R_naso*_26	S8	-56.99	4.81	Hap9	ROM 117594	EU096960
*R_naso*_31	S34	-58.72	4.73	Hap9	ROM_108965	EF080673
*R_naso*_33	S34	-58.72	4.73	Hap9	ROM_108977	EF080675
*R_naso*_41	S6	-56.9	4.53	Hap9	ROM_117106	JF447724
*R_naso*_45	S19	-58.52	4.28	Hap9	ROM_111568	JF455672
*R_naso*_52	S27	-59.02	4.75	Hap9	ROM_109220	JF455679
*R_naso*_55	S1	-59.08	4.53	Hap9	ROM_109066	JF455682
*R_naso*_62	S11	-59.53	2.82	Hap9	ROM_119038	JF455689
*R_naso*_69	S25	-58.22	6.5	Hap9	ROM_113779	JF455696
*R_naso*_73	S25	-58.22	6.5	Hap9	ROM_112618	JF455700
*R_naso*_96	S20	-58.83	1.53	Hap9	ROM_106688	JF455727
*R_naso*_14	S12	-82.25	8.35	Hap10	20120716_33	MG191904
*R_naso*_15	S12	-82.25	8.35	Hap11	20120716_31	MG191888
*R_naso*_16	S12	-82.25	8.35	Hap11	20120716_26	MG191880
*R_naso*_17	S28	-80.09	9.23	Hap12	20120815_217	MG191856
*R_naso*_18	S4	-80.03	9.22	Hap13	20120816_175	MG191846
*R_naso*_19	S12	-82.25	8.35	Hap13	20120716_27	MG191845
*R_naso*_20	S24	-88.65	17.76	Hap14	BCBF-203	JF499035
*R_naso*_21	S24	-88.65	17.76	Hap14	BCBF-202	JF499034
*R_naso*_22	S24	-88.65	17.76	Hap14	BCBF-201	JF499033
*R_naso*_23	S24	-88.65	17.76	Hap14	BCBF-200	JF499032
*R_naso*_24	S3	-76.43	-0.69	Hap15	ROM 118841	JF449155
*R_naso*_25	S5	-56.78	4.85	Hap16	ROM 117166	EU096962
*R_naso*_27	S8	-56.99	4.81	Hap16	ROM 117600	EU096956
*R_naso*_29	S7	-57.04	4.48	Hap16	ROM 116888	JF447725
*R_naso*_34	S34	-58.72	4.73	Hap16	ROM_108980	EF080676
*R_naso*_35	S5	-56.78	4.85	Hap16	ROM_117678	EU096954
*R_naso*_39	S5	-56.78	4.85	Hap16	ROM_117165	EU096961
*R_naso*_42	S7	-57.04	4.48	Hap16	ROM_116887	JF447726
*R_naso*_44	S7	-57.04	4.48	Hap16	ROM_116918	JF447728
*R_naso*_47	S19	-58.52	4.28	Hap16	ROM_111567	JF455674
*R_naso*_54	S1	-59.08	4.53	Hap16	ROM_109183	JF455681
*R_naso*_59	S34	-58.72	4.73	Hap16	ROM_108979	JF455686
*R_naso*_65	S11	-59.53	2.82	Hap16	ROM_119093	JF455692

*R_naso*_67	S15	-58.68	4.67	Hap16	ROM_115732	JF455694
*R_naso*_72	S9	-58.22	6.5	Hap16	ROM_112582	JF455699
*R_naso*_75	S32	-58.92	4.38	Hap16	ROM_111733	JF455702
*R_naso*_81	S23	-60.65	6.05	Hap16	ROM_F39757	JF455709
*R_naso*_84	S23	-60.65	6.05	Hap16	ROM_108103	JF455713
*R_naso*_92	S17	-58.85	4.73	Hap16	ROM_107302	JF455722
*R_naso*_97	S23	-60.65	6.05	Hap16	ROM_108099	JF455728
*R_naso*_99	S23	-60.65	6.05	Hap16	ROM_108100	JF455730
*R_naso*_102	S31	-59.1	3.92	Hap16	ROM_98094	JF455733
*R_naso*_105	S26	-57.3	5.17	Hap16	ROM_100445	JF455737
*R_naso*_109	S7	-57.04	4.48	Hap16	ROM_116965	JQ601313
*R_naso*_28	S5	-56.78	4.85	Hap17	ROM 117685	EU096955
*R_naso*_30	S34	-58.72	4.73	Hap18	ROM_108952	EF080672
*R_naso*_66	S15	-58.68	4.68	Hap18	ROM_115733	JF455693
*R_naso*_32	S34	-58.72	4.73	Hap19	ROM_108976	EF080674
*R_naso*_56	S27	-59.02	4.75	Hap19	ROM_109211	JF455683
*R_naso*_60	S34	-58.72	4.73	Hap19	ROM_108978	JF455687
*R_naso*_101	S33	-59.23	7.67	Hap19	ROM_98796	JF455732
*R_naso*_36	S5	-56.78	4.85	Hap20	ROM_117164	EU096957
*R_naso*_43	S7	-57.04	4.48	Hap20	ROM_116919	JF447727
*R_naso*_37	S5	-56.78	4.85	Hap21	ROM_117163	EU096958
*R_naso*_40	S6	-56.9	4.53	Hap22	ROM_117095	JF447723
*R_naso*_46	S25	-58.22	6.5	Hap23	ROM_112645	JF455673
*R_naso*_48	S25	-58.22	6.5	Hap23	ROM_112624	JF455675
*R_naso*_49	S25	-58.22	6.5	Hap24	ROM_112622	JF455676
*R_naso*_76	S25	-58.22	6.5	Hap24	ROM_112623	JF455703
*R_naso*_50	S34	-58.72	4.73	Hap25	ROM_108966	JF455677
*R_naso*_51	S1	-59.09	4.53	Hap26	ROM_109152	JF455678
*R_naso*_53	S1	-59.09	4.53	Hap27	ROM_109146	JF455680
*R_naso*_58	S1	-59.09	4.53	Hap27	ROM_109205	JF455685
*R_naso*_57	S1	-59.09	4.53	Hap28	ROM_109206	JF455684
*R_naso*_61	S11	-59.53	2.82	Hap29	ROM_119039	JF455688
*R_naso*_63	S15	-58.68	4.68	Hap30	ROM_115672	JF455690
*R_naso*_64	S11	-59.53	2.82	Hap31	ROM_119129	JF455691
*R_naso*_70	S25	-58.22	6.5	Hap32	ROM_113778	JF455697
*R_naso*_74	S19	-58.52	4.28	Hap33	ROM_111610	JF455701
*R_naso*_88	S10	-58.77	1.37	Hap33	ROM_106607	JF455717
*R_naso*_91	S20	-58.83	1.53	Hap33	ROM_106712	JF455721
*R_naso*_103	S31	-59.1	3.92	Hap33	ROM_98095	JF455734
*R_naso*_77	S19	-58.52	4.28	Hap34	ROM_111595	JF455705
*R_naso*_78	S13	-58.63	1.58	Hap35	ROM_106729	JF455706
*R_naso*_79	S17	-58.85	4.73	Hap36	ROM_107370	JF455707
*R_naso*_80	S13	-58.63	1.58	Hap37	ROM_106730	JF455708
*R_naso*_82	S10	-58.77	1.37	Hap38	ROM_106637	JF455711
*R_naso*_83	S13	-58.63	1.58	Hap39	ROM_106731	JF455712
*R_naso*_85	S10	-58.77	1.37	Hap40	ROM_106638	JF455714
*R_naso*_87	S14	-58.63	1.65	Hap41	ROM_106552	JF455716
*R_naso*_89	S17	-58.85	4.73	Hap42	ROM_107265	JF455719
*R_naso*_90	S18	-58.72	4.63	Hap42	ROM_107125	JF455720

*R_naso*_94	S17	-58.85	4.73	Hap43	ROM_107404	JF455724
*R_naso*_95	S10	-58.77	1.37	Hap44	ROM_106584	JF455726
*R_naso*_100	S31	-59.1	3.92	Hap45	ROM_98093	JF455731
*R_naso*_104	S2	-58.87	4.38	Hap46	ROM_98016	JF455735
*R_naso*_106	S22	-59.72	3.27	Hap47	ROM_97821	JF455740
*R_naso*_107	S16	-58.91	4.25	Hap48	ROM_119819	JF459272
*R_naso*_108	S16	-58.91	4.25	Hap48	ROM_119801	JF459273
