# Supplementary material for: Systematics and phylogeography of bats of the genus Rhynchonycteris (Chiroptera: Emballonuridae): Integrating molecular phylogenetics, ecological niche modeling and morphometric data
Source: PLoS One. 2023 May 4;18(5):e0285271. doi: 10.1371/journal.pone.0285271 (PMC10159116; doi:10.1371/journal.pone.0285271)
Supplement: S3 Table — Values below the diagonal are distances. Values above the diagonal are the p values. (PDF) [file pone.0285271.s006.pdf]

	Belize (Hap14)	Panamá (Hap10, Hap11, Hap12 and Hap13)	Cis-Andean
Belize (Hap14)		0.01	0.00
Panamá (Hap10, Hap11, Hap12 and Hap13)	0.83		0.00
Cis-Andean	0.92	0.91	
