# Supplementary material for: Systematics and phylogeography of bats of the genus Rhynchonycteris (Chiroptera: Emballonuridae): Integrating molecular phylogenetics, ecological niche modeling and morphometric data
Source: PLoS One. 2023 May 4;18(5):e0285271. doi: 10.1371/journal.pone.0285271 (PMC10159116; doi:10.1371/journal.pone.0285271)
Supplement: S4 Table — The numbers highlighted in gray refer to the level of sequence divergence within groups. (PDF) [file pone.0285271.s007.pdf]

	1	2	3	4	5	6	7	8	9	10	11	12	13	14	15	16	17	18	19	20	21
1. *B. io*	0.00 ± 0.00																				
2. *B. plicata*	20.32 ± 2.11	0.40 ± 0.19																			
3. *Ce. maximiliani*	28.04 ± 2.55	22.94 ± 2.24	0.41 ± 0.20																		
4. *Co. brevirostris*	26.24 ± 2.31	25.40 ± 2.24	21.29 ± 1.96	5.74 ± 0.84																	
5. *Cy. alecto*	21.94 ± 2.10	22.32 ± 2.22	21.61 ± 2.10	25.23 ± 2.23	3.67 ± 0.66																
6. *D. albus*	22.38 ± 2.09	20.77 ± 2.00	25.31 ± 2.34	25.13 ± 2.20	20.50 ± 2.01	4.01 ± 0.84															
7. *D. isabellus*	25.42 ± 2.38	23.50 ± 2.31	22.85 ± 2.20	24.31 ± 2.27	20.01 ± 2.05	14.98 ± 1.67	0.31 ± 0.15														
8. *P. kappleri*	24.16 ± 2.30	22.03 ± 2.09	19.21 ± 1.90	24.15 ± 2.15	21.10 ± 2.06	22.04 ± 2.07	23.86 ± 2.20	0.00 ± 0.00													
9. *P. leucoptera*	24.23 ± 2.38	21.66 ± 2.11	23.70 ± 2.27	23.28 ± 2.17	23.56 ± 2.29	23.49 ± 2.27	23.71 ± 2.37	19.79 ± 1.97	1.06 ± 0.33												
10. *P. macrotis*	22.46 ± 2.13	20.24 ± 1.91	18.95 ± 1.86	21.79 ± 1.98	23.03 ± 2.11	20.84 ± 1.94	23.34 ± 2.13	8.11 ± 1.10	20.29 ± 2.00	2.98 ± 0.40											
11. *P. pallidoptera*	22.03 ± 2.17	20.75 ± 2.07	21.70 ± 2.09	22.87 ± 2.14	19.74 ± 2.01	20.42 ± 2.01	20.06 ± 2.03	14.74 ± 1.61	20.50 ± 2.12	15.40 ± 1.64	NA										
12. *P. trinitatis*	24.01 ± 2.33	21.20 ± 2.12	19.13 ± 1.90	23.97 ± 2.22	21.40 ± 1.99	21.76 ± 2.04	23.09 ± 2.14	7.89 ± 1.15	19.81 ± 2.02	7.08 ± 1.03	15.53 ± 1.72	0.22 ± 0.13									
13. *R. naso* cis-Andean	22.85 ± 2.21	21.17 ± 2.09	23.90 ± 2.26	26.23 ± 2.32	20.80 ± 1.96	23.83 ± 2.13	23.71 ± 2.21	20.93 ± 2.02	22.78 ± 2.19	22.28 ± 2.05	20.73 ± 2.06	19.51 ± 1.93	0.88 ± 0.21								
14. *R. naso* trans-Andean	21.75 ± 2.11	19.96 ± 1.97	23.51 ± 2.17	26.23 ± 2.26	21.37 ± 2.00	23.22 ± 2.13	24.71 ± 2.30	20.69 ± 1.99	23.06 ± 2.25	21.31 ± 1.95	21.67 ± 2.04	20.82 ± 2.00	11.04 ± 1.37	1.97 ± 0.39							
15. *S. bilineata*	22.82 ± 2.05	20.48 ± 1.83	18.98 ± 1.81	22.20 ± 1.85	20.57 ± 1.91	22.82 ± 1.95	24.27 ± 2.17	20.07 ± 1.82	19.23 ± 1.80	21.18 ± 1.87	19.27 ± 1.79	21.58 ± 1.91	19.49 ± 1.80	20.56 ± 1.88	5.96 ± 0.66						
16. *S. canescens*	26.01 ± 2.44	26.62 ± 2.44	21.34 ± 2.08	22.17 ± 2.08	24.44 ± 2.32	22.84 ± 2.19	23.44 ± 2.30	23.23 ± 2.28	21.52 ± 2.10	26.63 ± 2.39	21.82 ± 2.21	24.32 ± 2.30	22.18 ± 2.15	22.59 ± 2.17	18.63 ± 1.85	2.09 ± 0.60					
17. *S. gymnura*	21.09 ± 2.08	21.36 ± 2.16	21.42 ± 2.27	24.64 ± 2.39	22.61 ± 2.25	22.58 ± 2.20	24.95 ± 2.48	21.44 ± 2.18	22.65 ± 2.21	21.03 ± 2.12	21.22 ± 2.10	21.25 ± 2.13	20.98 ± 2.10	23.11 ± 2.23	16.04 ± 1.67	19.66 ± 2.12	0.13 ± 0.12				
18. *S. leptura*	22.98 ± 2.14	22.88 ± 2.20	21.10 ± 2.04	24.34 ± 2.13	20.18 ± 1.95	21.31 ± 2.04	21.78 ± 2.13	21.85 ± 2.08	22.89 ± 2.19	23.99 ± 2.10	20.41 ± 1.96	25.36 ± 2.33	20.29 ± 1.99	21.40 ± 2.04	15.67 ± 1.52	19.97 ± 2.03	17.80 ± 1.98	1.15 ± 0.21			
19. *E. beccarrii*	27.52 ± 2.95	24.53 ± 2.75	23.90 ± 2.64	25.50 ± 2.72	25.91 ± 2.79	25.53 ± 2.78	28.32 ± 3.05	23.89 ± 2.51	24.66 ± 2.72	20.59 ± 2.15	24.53 ± 2.72	23.47 ± 2.53	22.98 ± 2.54	23.24 ± 2.52	23.15 ± 2.34	27.98 ± 2.98	25.47 ± 2.75	23.81 ± 2.56	4.20 ± 1.17		
20. *E. raffrayana*	26.57 ± 2.54	26.67 ± 2.55	24.54 ± 2.38	27.93 ± 2.54	23.90 ± 2.34	23.22 ± 2.21	25.89 ± 2.46	24.80 ± 2.36	26.45 ± 2.53	24.25 ± 2.24	23.92 ± 2.28	24.93 ± 2.38	25.15 ± 2.41	23.88 ± 2.24	24.87 ± 2.27	23.91 ± 2.32	23.30 ± 2.29	23.04 ± 2.18	23.82 ± 2.55	8.90 ± 1.37	
21. *T. longimanus*	23.78 ± 2.30	25.06 ± 2.30	27.61 ± 2.33	25.21 ± 2.22	23.47 ± 2.13	26.54 ± 2.37	27.63 ± 2.41	24.21 ± 2.22	25.97 ± 2.35	26.90 ± 2.32	25.06 ± 2.29	25.51 ± 2.23	25.54 ± 2.29	23.69 ± 2.24	24.61 ± 2.14	26.56 ± 2.46	26.45 ± 2.38	24.03 ± 2.16	29.99 ± 3.07	27.24 ± 2.57	NA
