# Supplementary material for: Systematics and phylogeography of bats of the genus Rhynchonycteris (Chiroptera: Emballonuridae): Integrating molecular phylogenetics, ecological niche modeling and morphometric data
Source: PLoS One. 2023 May 4;18(5):e0285271. doi: 10.1371/journal.pone.0285271 (PMC10159116; doi:10.1371/journal.pone.0285271)
Supplement: S5 Table — The numbers highlighted in gray refer to the level of sequence divergence within groups. (PDF) [file pone.0285271.s008.pdf]

	1	2	3	4	5	6	7	8	9	10	11	12	13	14	15	16	17	18	19	20	21
1. *B. io*	0.00 ± 0.00																				
2. *B. plicata*	17.10 ± 1.47	0.40 ± 0.18																			
3. *Ce. maximiliani*	22.62 ± 1.61	19.32 ± 1.56	0.41 ± 0.20																		
4. *Co. brevirostris*	21.47 ± 1.52	20.93 ± 1.50	18.31 ± 1.43	5.31 ± 0.71																	
5. *Cy. alecto*	18.62 ± 1.50	18.74 ± 1.55	18.40 ± 1.50	20.93 ± 1.51	3.49 ± 0.60																
6. *D. albus*	19.00 ± 1.49	17.86 ± 1.46	21.03 ± 1.57	20.96 ± 1.51	17.41 ± 1.43	3.85 ± 0.77															
7. *D. isabellus*	21.03 ± 1.60	19.64 ± 1.58	19.46 ± 1.56	20.39 ± 1.57	17.07 ± 1.47	13.27 ± 1.29	0.30 ± 0.15														
8. *P. kappleri*	20.12 ± 1.57	18.70 ± 1.49	16.69 ± 1.41	20.22 ± 1.49	18.07 ± 1.49	18.71 ± 1.46	20.02 ± 1.53	0.00 ± 0.00													
9. *P. leucoptera*	20.07 ± 1.60	18.37 ± 1.49	19.84 ± 1.56	19.47 ± 1.50	19.68 ± 1.57	19.72 ± 1.57	19.84 ± 1.63	16.96 ± 1.43	1.04 ± 0.32												
10. *P. macrotis*	19.01 ± 1.51	17.46 ± 1.40	16.53 ± 1.40	18.59 ± 1.44	19.42 ± 1.49	17.93 ± 1.42	19.68 ± 1.50	7.56 ± 0.95	17.33 ± 1.44	2.77 ± 0.34											
11. *P. pallidoptera*	18.76 ± 1.55	17.83 ± 1.51	18.52 ± 1.50	19.36 ± 1.51	17.03 ± 1.48	17.63 ± 1.48	17.36 ± 1.51	13.06 ± 1.26	17.48 ± 1.52	13.57 ± 1.27	NA										
12. *P. trinitatis*	20.07 ± 1.60	18.14 ± 1.53	16.67 ± 1.42	20.06 ± 1.53	18.30 ± 1.45	18.56 ± 1.46	19.51 ± 1.50	7.36 ± 1.00	16.98 ± 1.46	6.63 ± 0.90	13.67 ± 1.32	0.22 ± 0.13									
13. *R. naso* cis-Andean	19.25 ± 1.54	18.08 ± 1.50	19.90 ± 1.52	21.66 ± 1.56	17.90 ± 1.43	20.10 ± 1.49	19.92 ± 1.54	17.91 ± 1.46	19.20 ± 1.54	18.91 ± 1.46	17.81 ± 1.50	16.96 ± 1.44	0.87 ± 0.20								
14. *R. naso* trans-Andean	18.55 ± 1.52	17.24 ± 1.44	19.66 ± 1.48	21.66 ± 1.52	18.29 ± 1.44	19.65 ± 1.50	20.60 ± 1.57	17.69 ± 1.44	19.44 ± 1.57	18.20 ± 1.40	18.41 ± 1.45	17.85 ± 1.45	9.97 ± 1.12	1.92 ± 0.36							
15. *S. bilineata*	19.14 ± 1.43	17.59 ± 1.33	16.50 ± 1.35	18.89 ± 1.33	17.70 ± 1.40	19.31 ± 1.38	20.27 ± 1.49	17.35 ± 1.35	16.69 ± 1.34	18.15 ± 1.36	16.76 ± 1.35	18.44 ± 1.38	16.79 ± 1.32	17.59 ± 1.37	5.53 ± 0.57						
16. *S. canescens*	21.41 ± 1.61	21.95 ± 1.62	18.22 ± 1.49	18.94 ± 1.49	20.57 ± 1.61	19.42 ± 1.55	19.84 ± 1.62	19.62 ± 1.59	18.36 ± 1.51	21.88 ± 1.58	18.66 ± 1.59	20.37 ± 1.59	18.80 ± 1.53	19.12 ± 1.53	16.07 ± 1.37	2.05 ± 0.57					
17. *S. gymnura*	18.01 ± 1.50	18.24 ± 1.55	18.32 ± 1.63	20.51 ± 1.62	19.20 ± 1.60	19.12 ± 1.55	20.75 ± 1.66	18.35 ± 1.57	19.10 ± 1.53	18.09 ± 1.55	18.18 ± 1.52	18.24 ± 1.55	18.02 ± 1.53	19.55 ± 1.58	14.12 ± 1.28	16.85 ± 1.54	0.13 ± 0.12				
18. *S. leptura*	19.33 ± 1.49	19.35 ± 1.55	17.97 ± 1.46	20.36 ± 1.47	17.48 ± 1.45	18.30 ± 1.47	18.63 ± 1.54	18.55 ± 1.47	19.25 ± 1.52	20.03 ± 1.45	17.55 ± 1.43	20.93 ± 1.55	17.33 ± 1.43	18.15 ± 1.45	13.78 ± 1.17	17.08 ± 1.46	15.51 ± 1.49	1.13 ± 0.20			
19. *E. beccarrii*	22.55 ± 1.93	20.60 ± 1.89	20.25 ± 1.86	21.26 ± 1.85	21.52 ± 1.89	21.22 ± 1.88	23.01 ± 1.95	20.07 ± 1.74	20.54 ± 1.85	17.83 ± 1.59	20.41 ± 1.83	19.87 ± 1.77	19.52 ± 1.80	19.72 ± 1.78	19.68 ± 1.67	23.03 ± 1.98	21.34 ± 1.88	20.17 ± 1.80	4.06 ± 1.09		
20. *E. raffrayana*	21.97 ± 1.71	22.04 ± 1.71	20.53 ± 1.63	22.85 ± 1.66	20.25 ± 1.66	19.76 ± 1.57	21.62 ± 1.69	20.67 ± 1.60	21.86 ± 1.69	20.31 ± 1.54	20.13 ± 1.59	20.75 ± 1.62	21.00 ± 1.65	20.13 ± 1.57	20.82 ± 1.56	20.27 ± 1.65	19.87 ± 1.64	19.56 ± 1.54	19.99 ± 1.76	8.19 ± 1.15	
21. *T. longimanus*	20.15 ± 1.63	21.08 ± 1.60	22.91 ± 1.57	21.24 ± 1.56	20.00 ± 1.53	22.09 ± 1.61	22.84 ± 1.62	20.55 ± 1.58	21.78 ± 1.64	22.39 ± 1.58	21.20 ± 1.61	21.45 ± 1.55	21.45 ± 1.58	20.16 ± 1.59	20.80 ± 1.51	22.20 ± 1.69	22.11 ± 1.64	20.46 ± 1.55	24.44 ± 1.98	22.70 ± 1.77	NA
