# Supplementary material for: Systematics and phylogeography of bats of the genus Rhynchonycteris (Chiroptera: Emballonuridae): Integrating molecular phylogenetics, ecological niche modeling and morphometric data
Source: PLoS One. 2023 May 4;18(5):e0285271. doi: 10.1371/journal.pone.0285271 (PMC10159116; doi:10.1371/journal.pone.0285271)
Supplement: S6 Table — (PDF) [file pone.0285271.s009.pdf]

Specie	Intraspecific variability	Standard error
Balantiopteryx io	0	0
Balantiopteryx plicata	0.00	0.00
Centronycteris maximiliani	0.00	0.00
Cormura brevirostris	0.06	0.01
Cyttarops alecto	0.04	0.01
Diclidurus albus	0.04	0.01
Diclidurus isabellus	0.00	0.00
Peropteryx kappleri	0.00	0.00
Peropteryx leucoptera	0.01	0.00
Peropteryx macrotis	0.03	0.00
Peropteryx trinitatis	0.00	0.00
Rhynchonycteris naso	0.03	0.00
Saccopteryx bilineata	0.06	0.01
Saccopteryx canescens	0.02	0.01
Saccopteryx gymnura	0.00	0.00
Saccopteryx leptura	0.01	0.00
Emballonura beccarrii	0.04	0.01
Emballonura raffrayana	0.09	0.01
